# T cell-mediated curation and restructuring of tumor tissue coordinates an effective immune response

**DOI:** 10.1016/j.celrep.2023.113494

**Published:** 2023-12-11

**Authors:** John W. Hickey, Maximillian Haist, Nina Horowitz, Chiara Caraccio, Yuqi Tan, Andrew J. Rech, Marc-Andrea Baertsch, Xavier Rovira-Clavé, Bokai Zhu, Gustavo Vazquez, Graham Barlow, Eran Agmon, Yury Goltsev, John B. Sunwoo, Markus Covert, Garry P. Nolan

**Affiliations:** 1 Department of Microbiology & Immunology, Stanford University School of Medicine, Stanford, CA 94305, USA; 2 Department of Pathology, Stanford University School of Medicine, Stanford, CA 94305, USA; 3 Department of Bioengineering, Stanford University, Stanford, CA 94305, USA; 4 Department of Pathology and Laboratory Medicine, Perelman School of Medicine, University of Pennsylvania, Philadelphia, PA 19104, USA; 5 Center for Cell Analysis and Modeling, University of Connecticut Health, Farmington, CT 06032, USA; 6 Department of Otolaryngology, Head and Neck Surgery, Stanford Cancer Institute, Institute for Stem Cell Biology and Regenerative Medicine, Stanford University School of Medicine, Stanford, CA 94305, USA

**Keywords:** Multiplexed tissue imaging, spatial biology, T cells, metabolism, cancer immunotherapy, CODEX, T cell therapy, T cell phenotype, melanoma, checkpoint inhibitor therapy

## Abstract

Antigen-specific T cells traffic to, are influenced by, and create unique cellular microenvironments. Here we characterize these microenvironments over time with multiplexed imaging in a melanoma model of adoptive T cell therapy and human patients with melanoma treated with checkpoint inhibitor therapy. Multicellular neighborhood analysis reveals dynamic immune cell infiltration and inflamed tumor cell neighborhoods associated with CD8^+^ T cells. T cell-focused analysis indicates T cells are found along a continuum of neighborhoods that reflect the progressive steps coordinating the anti-tumor immune response. More effective anti-tumor immune responses are characterized by inflamed tumor-T cell neighborhoods, flanked by dense immune infiltration neighborhoods. Conversely, ineffective T cell therapies express anti-inflammatory cytokines, resulting in regulatory neighborhoods, spatially disrupting productive T cell-immune and -tumor interactions. Our study provides *in situ* mechanistic insights into temporal tumor microenvironment changes, cell interactions critical for response, and spatial correlates of immunotherapy outcomes, informing cellular therapy evaluation and engineering.

## Highlights

• CODEX multiplexed imaging of human and mouse, immunotherapy-treated melanomas
• Tumor-specific T cells cause inflamed tumor phenotype and unique cell neighborhoods
• T cell phenotype influences capacity to make effective tumor-killing neighborhoods
• Potent therapy creates larger, linked areas of inflamed tumor and immune infiltrate

## Graphical abstract

**Figure:**
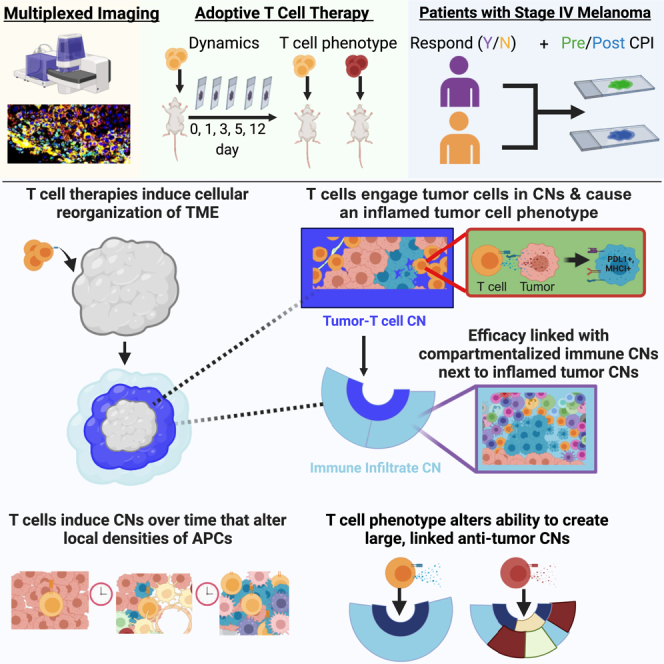

## Teaser

Hickey et al. discovered that tumor-specific T cells induce tumor inflammation and establish progressive inflammatory environments. Effective therapies exhibit compartmentalized immune infiltration, co-located with T cell and inflamed tumor zones, emphasizing the need for therapeutic T cells to be designed to curate local and distal microenvironments.

## Introduction

T cells mediate antigen-specific responses that are essential for efficient immune surveillance in the human body. Consequently, they encounter and must be able to effectively respond within a wide number of tissue microenvironments. An outstanding question is how antigen-specific T cells must manipulate the local tissue microenvironment to accomplish effector function coupled to the extent to which we can alter T cell biology to overcome mechanisms of tumor immune evasion to accomplish positive therapeutic outcomes.

Compared with small molecule and protein-based drugs, T cell-based immunotherapies offer substantial benefits in terms of antigen specificity, proliferation, and memory. Particularly, immunotherapy using checkpoint inhibitors (CPIs) has had incredible clinical impacts in the last decade and is now used as single agents or combination therapies in about 50 cancer types.^1^ Despite the remarkable clinical efficacy that was observed in some patients, the majority of patients do not respond to CPI therapy or develop secondary acquired resistance.^2^ Similarly, T cell therapies have resulted in impressive clinical outcomes^3^^,^^4^^,^^5^^,^^6^; yet, lack of efficacy and systemic toxicities have limited broader application of these therapies to solid tumors.^7^^,^^8^

Advances in synthetic biology^8^^,^^9^ or modulation of T cell phenotype^10^^,^^11^^,^^12^^,^^13^^,^^14^ promise to hold solutions to challenges of toxicity, tumor infiltration, and modulation of the tumor microenvironment facing immune cell therapies. Analyses using single-cell technologies have shown that improved function results from enhanced capacity for self-renewal, killing, and resistance to cancer cell-mediated immunosuppression.^15^^,^^16^^,^^17^^,^^18^^,^^19^^,^^20^ However, our understanding remains limited of how T cells enter tumor microtissue and how such cellular structures react to perturbations initiated by T cell therapies.

Indeed, the challenges and outstanding questions in targeting certain classes of solid cancers are spatially related: trafficking, tumor antigen expression, and spatial co-enrichment with stimulating or inhibiting immune cells will likely drive different outcomes to therapy. Determining how these spatial relationships and multicellular neighborhoods are altered based on T cell phenotype will further reveal the molecular and cellular mechanisms driving therapeutic efficacy. Ultimately, a dataset that links spatial correlates of therapeutic efficacy such as cell-cell interactions and cellular neighborhoods, and the dynamic changes of tissue microenvironments following therapeutic intervention, to T cell biology will improve our capacity to better engineer cell therapies.

To evaluate how therapeutic T cells influence the dynamic, cellular anti-tumor response and how perturbing T cell phenotype impacts the tumor microenvironment, we applied recently developed multiplexed imaging technologies that enable single-cell analysis of more than 50 targets at once in tissue sections.^21^^,^^22^ We applied these to analyze tumors in a syngeneic, murine B16F10 model of antigen-specific T cell therapy.^5^^,^^11^^,^^16^^,^^23^^,^^24^^,^^25^ We both study tumors across time points and also with differential phenotypes. Similarly, we investigated the dynamic changes of the tumor microenvironments of responding and non-responding metastatic melanoma patients before and after initiation of CPI therapy. In summary, we show distinct developmental pathways for the formation of active, higher-order spatial biology that is correlated with success of T cell-based immunotherapies. Such findings can guide development of strategies for screening of T cells prior to transfer to ensure optimal efficacy and for engineering of T cell therapies to yield more productive anti-tumor immune responses.

## Results

### CODEX multiplexed imaging shows T cell therapy induces dynamic immune cell infiltration and tumor inflammation

**Figure 1:**
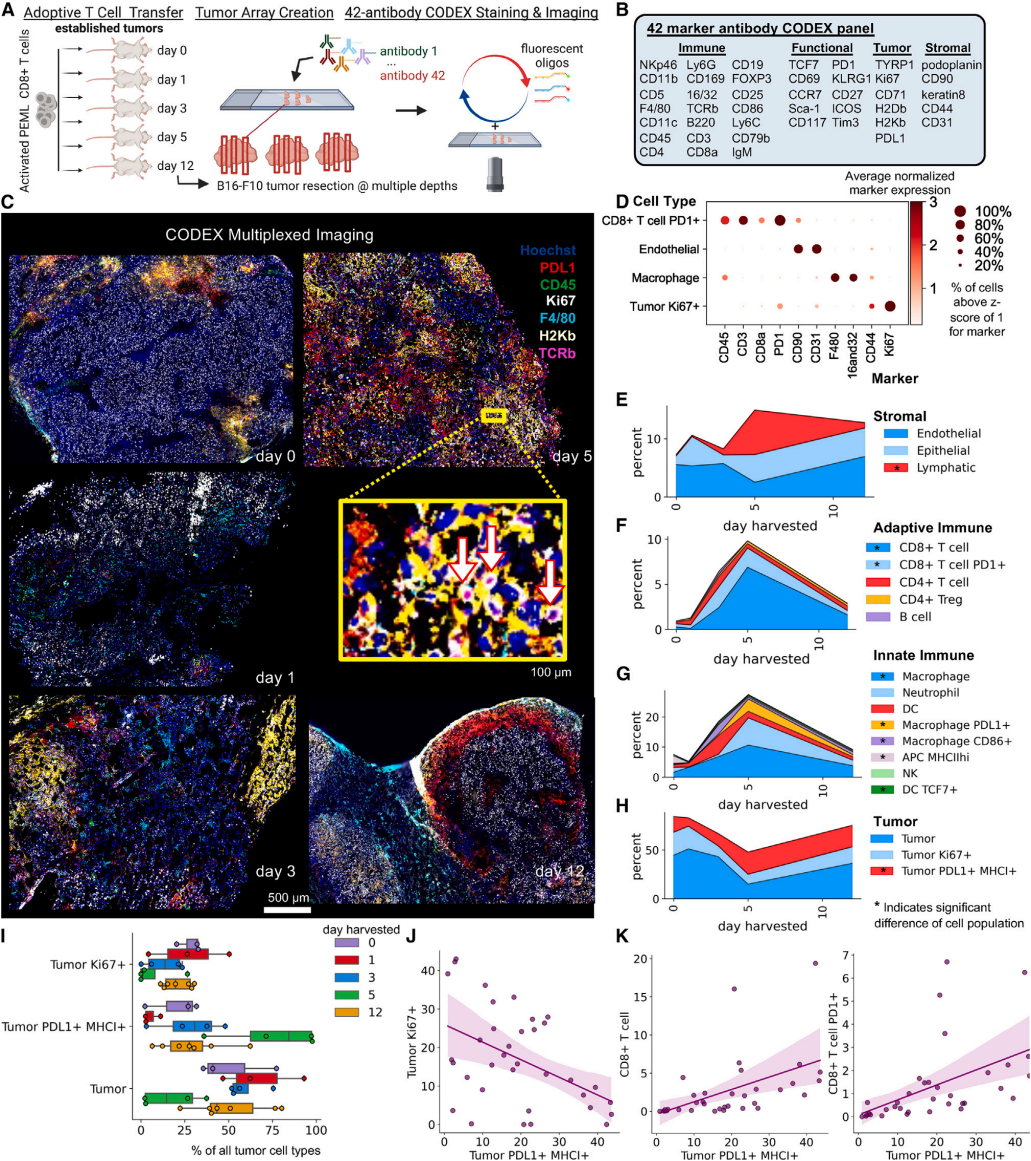
CODEX multiplexed imaging of T cell therapy shows dynamic induction of immune cell infiltration and tumor cell inflammation (A) Experimental layout for T cell therapy model and CODEX multiplexed imaging. (B) Forty-two-antibody panel used for CODEX multiplexed imaging. (C) Representative CODEX images of B16-F10 tumors from mice treated on day 10 with activated PMEL therapeutic T cells and harvested 0, 1, 3, 5, or 12 days following treatment. Scale bars, 250 μm, and the zoomed box is 100 μm long. (D) Normalized expression of 10 proteins in four of 23 identified cell types. (E–H) Percentages of indicated cells types out of total cells in (E) stromal, (F) adaptive immune, (G) innate immune, and (H) tumor populations over time (plots show averages from three to seven replicates, ^∗^ indicates p value < 0.05, one-way ANOVA test). (I) Percentages of tumor cell subtypes normalized to total number of tumor cells for each sample (n = 3–7). The boxplots show the median (center line), 25th to 75th percentile (box limits), minimum and maximum values (whiskers), and outliers (points outside 1.5× the interquartile range). (J) Percentage of Ki67^+^ tumor cells and (K) percentage of CD8^+^ PD1^+^ T versus percentage of PDL1^+^ H2Kb^+^ tumor cells per sample.

To evaluate the coordination of efficient immune responses along with antigen-specific T cell and tumor interactions, we chose an established, antigen-specific, syngeneic murine melanoma model of adoptive T cell therapy.^5^^,^^16^^,^^23^^,^^26^ Activated PMEL CD8^+^ T cells were transferred intravenously into mice with established, palpable B16-F10 tumors. Tumors were imaged with CO-Detection by indEXing (CODEX) multiplexed imaging^27^^,^^28^^,^^29^ with a 42-plex antibody panel at 0-, 1-, 3-, 5-, and 12-days post-treatment (Figures 1A and 1B, n = 3–7).

On days 0 and 1, a large proportion of tumor cells were proliferating, and there was little immune infiltration (Figure 1C). By day 3, there were multiple areas of the tumor with upregulated major histocompatibility complex class I (MHCI), colocalized in areas with increased immune cell infiltration. MHCI upregulation further increased on day 5 as were levels of PDL1 and immune cells, such as CD8^+^ T cells. By day 12, there was still evidence of inflammation but had become increasingly compartmentalized (Figure 1C).

These trends are mirrored by cell-type percentages (Figures 1D, S1A, and S1B) that varied more across conditions than multiple depths (Figure S1C), indicating that the tumor immune response is largely conserved throughout an individual tumor. Within the stromal compartment of tumors from T cell-treated mice, the percentage of endothelial cells was decreased on day 5, whereas the lymphatic cell percentage increased (Figures 1E and S2), supporting an important dynamic balance of vasculature and lymphatics in tumor growth rates.^30^^,^^31^^,^^32^

Percentages of adaptive immune cells increased over the first 5 days (Figure 1F). Particularly CD8^+^ T cells increased from ∼0.5% to nearly 5% of all cells at day 3 and 10% at day 5. In contrast, CD4^+^ T cell subsets remained at approximately 1% of all cells, though CD4^+^ regulatory T cells (Tregs) increased between day 3 and day 12. Interestingly, by day 12 we observed a decrease in percentages across all T cell subtypes, reflecting the aggressive growth kinetics of B16F10 melanoma.

Innate immune cells made up the majority of immune cell types on day 1, with nearly a 10 to 1 ratio to adaptive immune cells, though this decreased to about 3 to 1 on days 3, 5, and 12 (Figure 1G). This indicates that therapeutic T cells either initially home to secondary lymphoid organs or require additional time (∼3 days) for expansion and stimulation from a small starting percentage within the tumor.

Changes in tumor cell phenotype match our qualitative observations with increasing inflammation over the 12-day period (Figures 1H and S2D). The percentage of tumor cells that express PDL1 and MHCI increased from less than 10% on day 1 to nearly 70% by day 5 post therapy (Figure 1I). Indeed, PDL1^+^ MHCI^+^ tumor cell percentages were negatively correlated with Ki67^+^ tumor cells across all sections and on each day, indicating a tumor phenotype conversion (Figure 1J, r = −0.81 Pearson correlation). On the other hand, percentages of PDL1^+^ MHCI^+^ tumor cells were positively correlated with percentages of CD8^+^ T cells (Figure 1K, all values r > 0.8). Thus, adoptive T cell therapy induces an anti-tumor immune response that induces a tumor inflammatory phenotype conversion and broad intratumoral immune infiltration.

### CD8^+^ T cells are associated with inflammatory tumor cell neighborhoods

**Figure 2:**
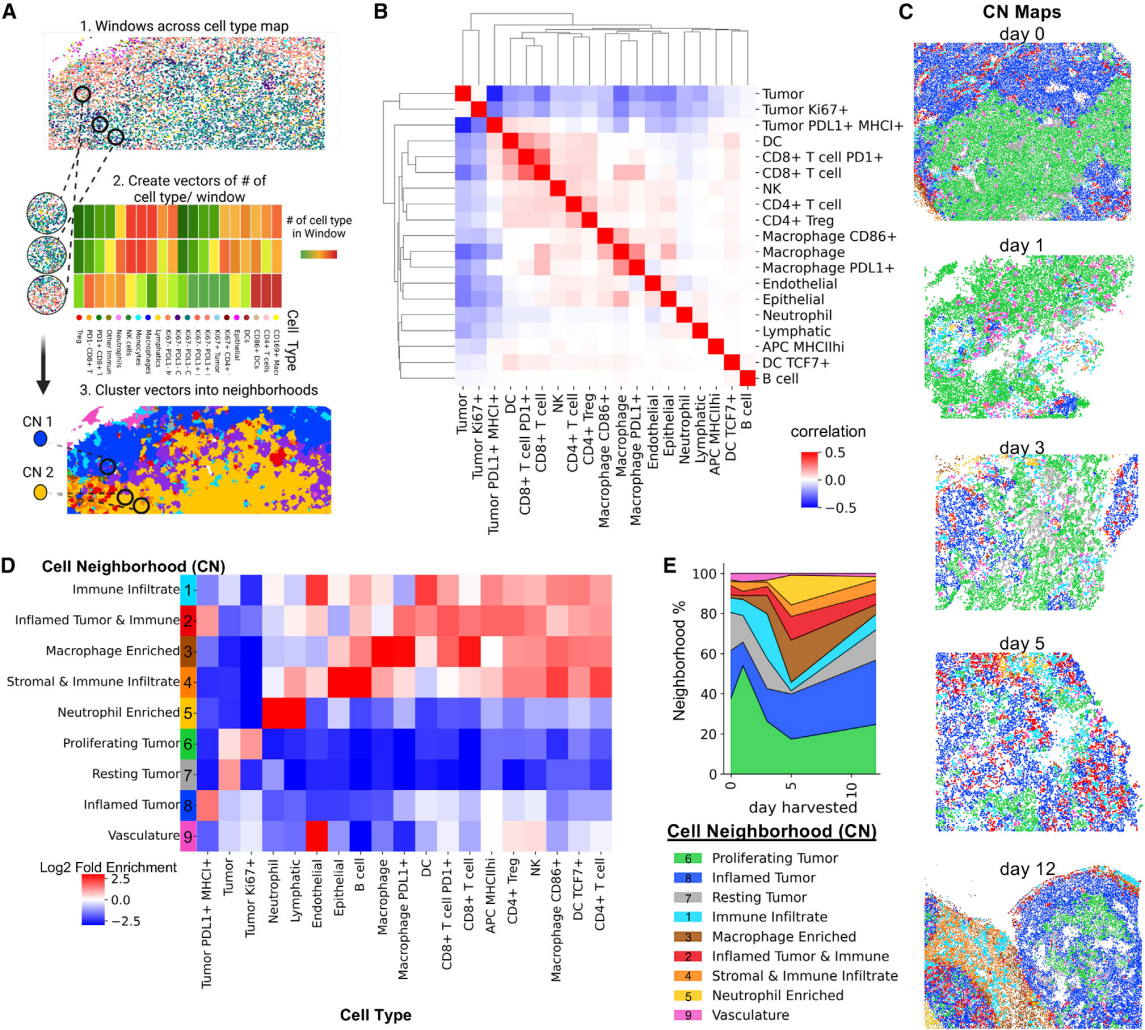
CD8^+^ T cells are associated with inflammatory tumor cell neighborhoods (A) Schematic of multicellular neighborhood analysis. (B) Correlation heatmap of cell-type abundance within cell neighbor windows. (C) Representative images of neighborhoods mapped to tumor tissues from days 0, 1, 3, 5, and 12 post-treatment. (D) Heatmap of fold enrichment of each cell type as compared with tissue averages in each of the neighborhoods identified. (E) Quantification of neighborhood percentage over time (plots show averages from three to seven replicates).

Because we qualitatively observed a non-uniform spatial distribution of cells (Figure 1C), we posited that spatial analysis of our defined cell types could classify conserved cellular microenvironments in the tumor. We used a method to extract each cell’s local microenvironment by cataloging its distinct cellular neighborhoods (CNs) (Figure 2A). Co-correlation indicated that CD8^+^ T cells tend to spatially co-cluster with dendritic cells (DCs) and also the tumor PDL1+ MHCI+ cells (Figure 2B). Since we observed multiple modules, we clustered into distinct CNs with unsupervised clustering,^33^ resulting in nine major CNs that could be visualized on the tissue (Figure 2C).

Four of the nine CNs are enriched with tumor cell types. Many of the tumor neighborhoods are more homogeneous and enriched only for a few cell types. For example, CN6 (*Proliferating Tumor*) is enriched for marker negative tumor cells and for Ki67^+^ tumor cells and depleted for all other cell types (Figure 2D). Similarly, CN7 (*Resting Tumor)* is enriched for marker negative tumor cells and is often found localized with CN6, interspersed occasionally by CN9 (*Vasculature*) (Figure 2C), and this finding agrees with our correlation modules (Figure 2B). These CNs decreased from days 1–5 but gained greater percentages by day 12 (Figure 2E). Thus, areas where the tumor is proliferating are compositionally devoid of immune cells and span large areas of immune deserts across the tumor (Figure 2C).

In contrast, PDL1^+^ MHCI^+^ tumor cells are enriched in CN8 and CN2 where CN2 contains an enrichment of the majority of immune cells including CD8^+^ T cells (Figure 2D). Areas of an inflamed tumor phenotype, indicated by the CN8 and CN2 are often found together, alongside other inflammatory CNs (1, 3, 4, 5) (Figure 2C) that are distinct organizations of immune and stromal cell populations not enriched for tumor cells (Figure 2D). These inflammatory CNs increased from days 1–5 but shrank in overall percentage by day 12, suggesting cooling of the tumor response by day 12 (Figure 2E). The spatial association of PDL1^+^ MHCI^+^ tumor cells and CD8^+^ T cells (Figure 2D) and the correlation between their frequencies (Figure 1K) indicates a role of the T cells in converting the tumor cell phenotype. In a separate study, we demonstrated the importance of tumor phenotype conversion for tumor control in multiscale modeling simulations of adoptive T cell therapies.^34^

### T cell-focused spatial analysis shows a time-linked continuum of cell neighborhoods

Since there was a time-dependent change in neighborhood composition of the tumor tissue after treatment with T cells (Figure 2E) but spatial patterning of neighborhoods remained relatively constant (Figure 2C), we hypothesized that a more granular look into the single-cell data could give clues to transition states of neighborhoods. Particularly, views into the ways CD8^+^ T cell environments change over time may guide the design of more effective T cell therapies.

**Figure 3:**
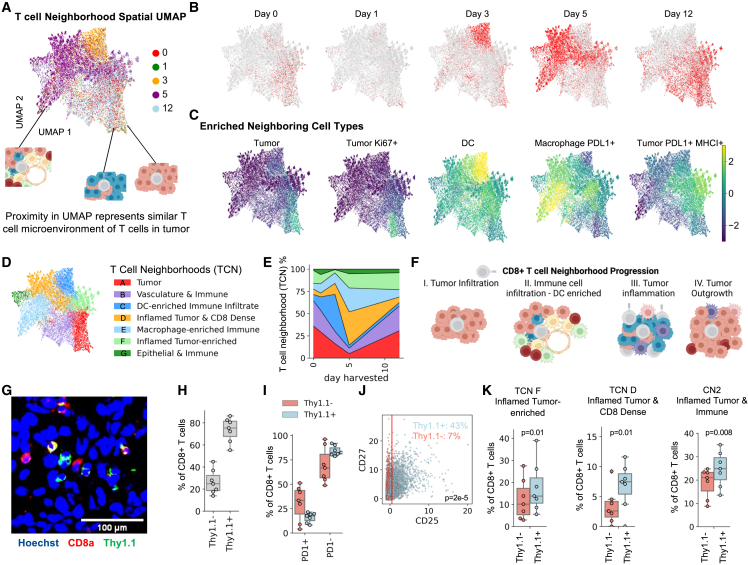
Newly developed T cell-focused spatial analysis showing that CD8^+^ T cells can be found along a continuum of cell neighborhoods representing steps of inflammation and tumor attack (A) T cell centric spatial UMAP encodes how similar the local microenvironment surrounding individual CD8^+^ T cells is to other CD8^+^ T cell microenvironments. (B and C) Spatial UMAP plots of T cell neighborhood composition vectors across all experiments with T cells from tumors harvested (B) each day highlighted in red. (C) Overlays of cell-type enrichment of each T cell microenvironment. (D and E) Seven T cell neighborhoods (TCNs) were found and (D) plotted on UMAP coordinates and (E) percentages of T cell neighborhoods present at each time point (plots show averages from three to seven replicates). (F) Illustration of dynamic response of the tumor microenvironment to T cell therapy. (G) Representative CODEX images of B16–F10 tumors harvested on day 12 after being treated with activated PMEL therapeutic T cells. Scale bar, 100 μm long. (H) Percent of total CD8^+^ T cells that are Thy1.1+/− from tumors harvested 12 days after adoptive T cell transfer treatment (n = 7). (I) Percent of CD8^+^ T cells that are positive for PD1 staining for either Thy1.1+/− cells from tumors harvested 12 days after adoptive T cell transfer treatment (n = 7). (J) Scatterplot of the normalized expression of CD25 and CD27 by either Thy1.1+ (blue) or Thy1.1− (pink) CD8^+^ T cells and gate indicating positivity for CD25. (K) Percent of CD8^+^ T cells that are positive for either TCN F or D and CN2 (n = 7, p value = 0.01, 0.01, and 0.008 calculated by two-sided t test). For H, I, and K the boxplots show the median (center line), 25th to 75th percentile (box limits), minimum and maximum values (whiskers), and outliers (points outside 1.5× the interquartile range).

To only look at the cellular environments surrounding CD8^+^ T cells, we developed a new version of CN analysis that is cell-type centric, with each point in the UMAP representing an individual CD8^+^ T cell found within the tumor microenvironment (Figure 3A). We identified cells that are co-enriched as neighbors with T cells from different timepoints to create a timeline of T cell neighborhood evolution (Figure 3B). On days 0 and 1, the few CD8^+^ T cells observed were mostly associated with marker negative and Ki67^+^ tumor cells (Figure 3C, panels 1 and 2). On day 3, T cells were also observed next to marker negative and Ki67^+^ tumor cells and endothelial cells, indicating initial T cell infiltration (Figure 3C, panels 1 and 2; Figure S3A). On day 3, T cells were also associated with lymphatic cells and other immune cells such as DCs, indicative of immune cell recruitment (Figure 3C, panel 3; Figure S3A). By day 5, T cells disengage with DCs and are enriched with higher levels of macrophages and found associated with PDL1^+^ MHCI^+^ tumor cells (Figure 3C, panels 4 and 5), an association that increased on day 12 (Figure 3C, panel 5).

We clustered these CD8^+^ T cell neighborhood windows and used cell-type enrichments within each cluster to define seven major T cell-specific neighborhoods (TCNs) (Figures 3D and S3B). Interestingly, all TCNs are found across all tumors that were taken at different time points post-treatment. However, each day is enriched in different proportions of TCNs, confirming changes in T cell tumor microenvironments over time. On days 0 and 1, the T cells were often associated with tumor (TCN A) and both vasculature (TCN B) (Figure 3E), indicating tumor infiltration processes (Figure 3F*i*). On day 3, T cells were found more in TCN C that was enriched with DCs and CD4^+^ T cells (Figure 3E), indicating local antigen presentation and activation (Figure 3F*ii*). In contrast, on day 5, T cells were not often observed within vasculature neighborhood (TCN B), which is supported by increased average distances between CD8^+^ T cells and endothelial cells over time (Figure S3C). Instead CD8^+^ T cells were found within areas of tumor cells characterized by an inflamed phenotype (TCN D, F) and with other immune cells (TCN E, G) (Figure 3E), indicating active tumor killing (Figure 3F*iii*). At day 12, the composition of TCNs largely mimics the compositions of days 0 and 1 (Figure 3E), indicating tumor growth that T cell responses are not able to keep up with (Figure 3F*iv*). Taken together, these data indicate that T cells could be found along a time-dependent continuum of neighborhoods, particularly with changes in antigen-presenting cell compositions.

To understand if there were any differences in the tumor microenvironment specifically for adoptively transferred cells, we stained T cells in CODEX multiplexed imaging experiments also with Thy1.1, which marks PMEL-derived CD8^+^ T cells (Figure 3G). At day 12, about 75% of the CD8^+^ T cells within the tumor were from adoptively transferred cells (Figure 3H). Surprisingly, adoptively transferred cells (Thy1.1^+^) had a decreased proportion of PD1^+^ cells than endogenous CD8^+^ T cells in the tumor microenvironment (Figure 3I). Additionally, there was a higher percentage of transferred cells that expressed higher levels of CD25, indicative of activation, recent antigen encounter, effector function, and proliferation (Figure 3J). Aligned with this phenotypic shift, we see adoptively transferred cells are more likely to be found in areas engaged with inflamed tumor cells (TCN F, D, and CN2) (Figures 3K and S4), indicating a preferential active anti-tumor response by transferred cells as compared with host-derived T cells.

### Metabolically treated T cells induce productive T cell and tumor neighborhoods flanked by neighborhoods of immune infiltrate

Having identified critical steps within the dynamic endogenous immune response to an antigen-specific T cell therapy for our B16F10 melanoma model, we next asked how manipulation of the T cell phenotype might modulate the tumor microenvironment.^11^^,^^18^^,^^19^^,^^20^^,^^26^^,^^35^^,^^36^ One study using this model showed that acetyl-CoA inhibited T cells were more effective at eradicating tumors than were cells activated only with gp100 and interleukin (IL)-2.^11^ We therefore activated the T cells with 2-hydroxycitrate (2HC), a metabolic inhibitor of acetyl-CoA production^11^ (further referred to as *2HC T cells*). We evaluated the T cell phenotype following culture, and *2HC T cells* were enriched for memory phenotypes, whereas *T cells* were enriched for effector and exhausted phenotypes (Figures S5A–S5E), in agreement with previous results.^11^ We treated tumor-bearing mice with activated *2HC T cells* or *T cells* and both T cell treatments provided control of the tumor, with 2HC providing slight increase in efficacy over conventionally activated T cells (Figures S5F and S5G). As opposed to Vodnala et al., we did not detect a complete reduction for the 2HC-treated T cell condition in the B16F10 model, which could be attributed to the aggressive nature of the B16F10 model.^11^

**Figure 4:**
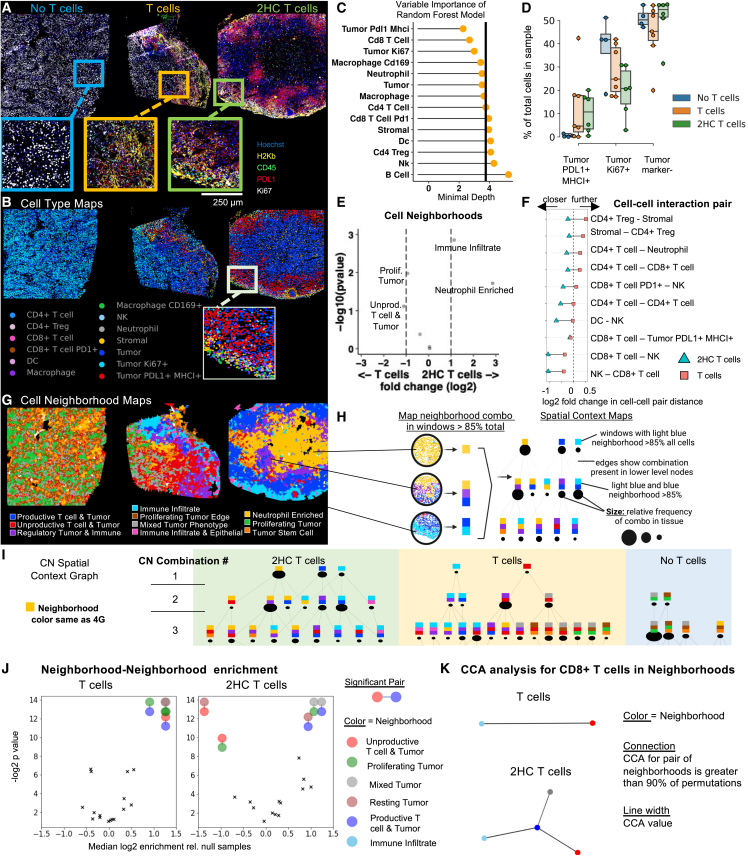
Metabolically treated T cells induce productive T cell and tumor neighborhoods flanked by neighborhoods of immune infiltrate (A) Representative CODEX images and (B) Cell-type maps of the treated and untreated tumors. Scale bar, 250 μm. (C) Minimal depth of cell-type percentages that differentiate treatment groups. (D) Tumor cell phenotypes measured by CODEX multiplexed imaging of tumors from controls (no T cells) and mice treated with T cells or 2HC T cells (n = 4–7 individual tumors from four independent experiments). The boxplots show the median (center line), 25th to 75th percentile (box limits), minimum and maximum values (whiskers), and outliers (points outside 1.5× the interquartile range). (E) Volcano plot of neighborhood percentage from CODEX imaging data for *2HC T cells* or *T cells* treated tumors (p value calculated from likelihood ratio test). (F) Selected significant cell-cell interaction pairs and compared log2 fold distance calculations compared with null distribution average. (G) Neighborhoods mapped back to tissue coordinates. (H) Schematic of spatial context map generation. (I) Spatial context maps where row shows the number of neighborhoods in combinations. (J) Significant two-combination neighborhood motifs enriched or depleted compared with null distribution of permuted samples. (K) Canonical correlation of CD8^+^ T cell frequencies across all neighborhoods. Linkages plotted indicate correlations found above permuted controls between sets of neighborhoods with color as shown in the key in (G).

Three days post-treatment, CODEX imaging of the tumors indicated immune infiltration (CD45), tumor inflammation (H2Kb, PDL1), and downregulation of tumor proliferation (Ki67) from both T cell-treated tumors compared to the control tumor not treated with T cells (referred to as *No T cells*) (Figures 4A and 4B, n = 4–7). Indeed, inflamed tumor cells (PDL1^+^ MHCI^+^), CD8^+^ T cells, and Ki67^+^ tumor cells were identified as the three most important cell-type percentages that could predict treatment group (Figure 4C, AUC = 0.95). In tumors that received either T cell treatment, there was an increase in PDL1^+^ MHCI^+^ tumor cells, whereas there were less than 2% inflamed tumor cells in the control tumors, associated with higher levels of CD8^+^ T cells (Figures 4D and S6). Moreover, tumors from mice treated with *2HC T cells* had a lower percentage of proliferating tumor cells than did either of the other conditions (Figure S6A). This further confirms that T cells can mediate tumor cell proliferation inhibition and induce tumor cell inflammation.

Characterization of the CNs for all three conditions revealed four tumor CNs and six immune-tumor CNs (Figure S7A) with similarities to those found in our dynamic analysis in Figure 2. One major difference was two CNs involving CD8^+^ T cells and inflamed tumor cells. One of these CNs was enriched for MHCI^+^ tumor cells, CD4^+^ T cells, Ki67^+^ CD4^+^ T cells, and NK cells (termed herein *Productive T cell & Tumor* CN). In contrast, the second neighborhood lacks these features and is enriched for Tregs and MHCI^−^ tumors (termed as the *Unproductive T cell & Tumor* CN). The tumors treated with *T cells* had a higher proportion of *Unproductive T cell & Tumor* CN compared with the tumors treated with *2HC T cells* (Figures 4E and S7B). Cell-cell interaction analysis also indicated that CD8^+^ T cells are closer to both NK cells and CD4^+^ T cells in tumors treated with *2HC T cells*, also having closer CD4^+^ T cell-CD4^+^ T cell associations (Figures 4F, S7C, and S7D). Similarly, we observed that CD4^+^ Tregs were closer to stromal cell types, which helps explain spatial segregation of the CD4^+^ Tregs away from *Productive T cell & Tumor* CNs in *2HC T cell*-treated tumors. Finally, CN analysis indicates *No T cells* had uniform spatial patterning of mostly tumor CNs (Figure 4G). In both tumors treated with T cells, there was increased regional density of immune-based CNs, and there were clear borders around CNs (Figure 4G). In tumors treated with *2HC T cells*, the *Productive T cell & Tumor* CN (blue) was often bordered by the *Immune Infiltrate* CN (light blue) (Figure 4G).

Multicellular neighborhood interfaces may indicate coordination of critical functions within the tumor.^21^ To quantify and formalize general observations about CN associations, we used spatial context analysis developed previously (Figure 4H).^37^ The tumors treated with *2HC T cells* had a greater proportion of compartmentalization than the other treatment groups (Figure 4I, row 1). Here, CNs such as *Productive T cell & Tumor* (blue) have high degrees of compartmentalization. This indicates that these T cells transform large areas of the tumor into regions with conserved cell-type composition of immune infiltrate and T cell-mediated tumor cell killing areas. In contrast, *No T cells* condition has a lack of coordinated structure with the majority in combinations of three or more CNs (Figures 4I and S8A).

The cases where a combination of two CNs make up at least 85% of a window are indicative of important interfaces and coordination of structures potentially necessary for function. In tumors treated with *2HC T cells*, there were higher proportions of the *Productive T cell & Tumor* (blue) and *Immune Infiltrate* (light blue) CN combination than in the other two groups (Figures 4I and S8B–S8D). In contrast, the *Productive T cell & Tumor* and *Unproductive T cell & Tumor* CN combination was prevalent in tumors treated with *T cells*.

We next evaluated CN interactions^37^ and in tumors treated with *T cells*. Here, we observed a significant association of the *Productive T cell & Tumor* neighborhood (blue) with the *Unproductive T cell & Tumor* (red) CN; whereas this motif was not detected in the tumors treated with *2HC T cells* (Figure 4J). In contrast, within the tumors treated with *2HC T cells*, we observed combinations of the *Productive T cell & Tumor* neighborhood (blue) with tumor-based CNs. Consequently, we observe this indicates the *Productive T cell & Tumor* CN plays an important role in the tumor response and is impacted by which CNs border it.

Canonical correlation analysis^33^ also revealed that frequencies of CD8^+^ T cells are differentially correlated along connections of the Immune Infiltrate and Productive T cell & Tumor CNs (Figure 4K). This demonstrates that spatial relationships between inflamed tumor CNs and the *Immune Infiltrate* neighborhood are also critical for CD8^+^ T cell function in the tumor*.* Overall, these data suggest that pairing of *Unproductive* and *Productive T cell & Tumor* CNs is undesirable, and that proximity of *Immune Infiltrate* CNs is critical for CD8^+^ T cell function.

### Acetyl-CoA pathway modulated T cells have a distinct capacity to shape coordinated immune infiltration

**Figure 5:**
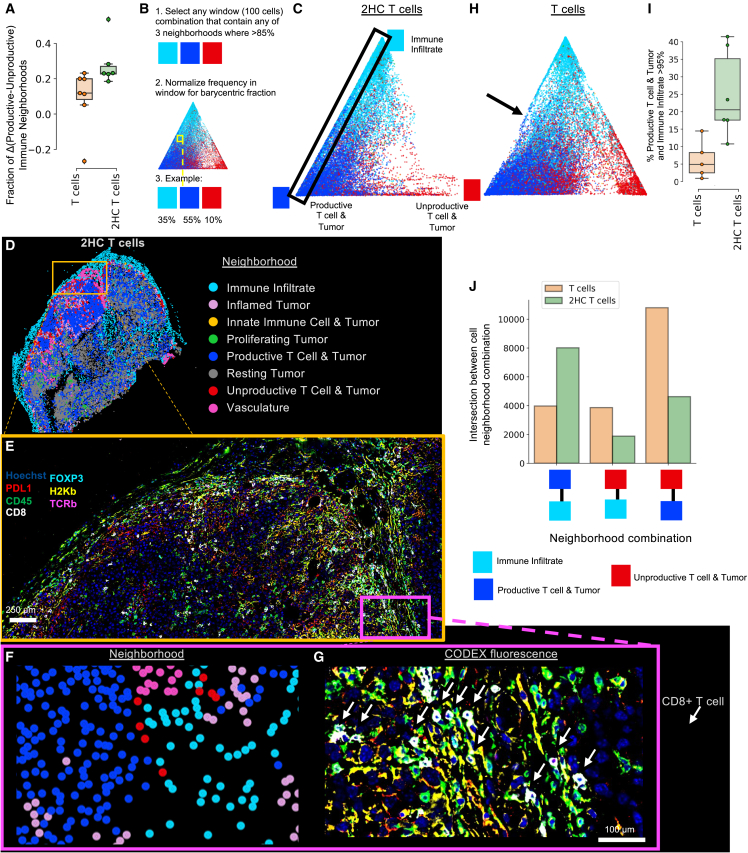
The phenotype of the therapeutic T cell influences its capacity to shape coordinated immune infiltration (A) Productive neighborhood fraction per sample (n = 6–7 individual tumors from four independent experiments). The boxplots show the median (center line), 25th to 75th percentile (box limits), minimum and maximum values (whiskers), and outliers (points outside 1.5× the interquartile range). (B) Graphic of generation of barycentric plots of window combinations. (C) Barycentric plot for tumors from mice treated with 2HC-activated T cells (cells from n = 6 individual tumors from four independent experiments). (D) Neighborhood map for a tumor from a mouse given 2HC-treated T cells. (E) Magnification of region in orange rectangle in (D) with six of the 42 CODEX fluorescent markers shown. Scale bar, 250 μm. (F and G) Magnification of region in magenta rectangle in (E) with both the (F) neighborhood and (G) CODEX fluorescent imaging overlay shown. White arrows indicate CD8^+^ T cells. Scale bar, 100 μm. (H) Barycentric plot of data from conventional-activated T cell treatment group (cells from n = 7 individual tumors from four independent experiments). (I) Quantification of percentage of windows that have 95% or greater of *Productive T cell & Tumor* and *Immune Infiltrate* neighborhoods (n = 5–6 individual tumors from four independent experiments, p < 0.05 from two-sided t test). (J) Intersection of the neighborhood instances quantified for defined neighborhood combinations.

We added the fractions of *Immune infiltrate* and *Productive T cell & Tumor* CNs and subtracted the fraction of the *Unproductive T cell & Tumor* CN for each condition. This value was higher for tumors treated with *2HC T cells* than for mice treated with *T cells* (Figure 5A, p < 0.05). This suggests that *2HC T cells* have greater propensity to create productive T cell tumor killing areas than do *T cells*.

We surmised that compositional differences when these CNs are found in the same area may be more informative than bulk averages. To quantify this, we measured the relative percentages of these three CNs in windows where they made up at least 85% of the CNs (Figure 5B)—or all possible combinations with these CNs from our spatial context map (Figure 4I). For tumors given *2HC T cells*, there was a high proportion of cells that fall in the interface of the *Immune Infiltrate* and *Productive T cell & Tumor* CNs (Figure 5C, black rectangle). This localized combination of CNs (blue, light blue) can be seen when CN labels are mapped to a tumor treated with *2HC T cells* (Figure 5D). Focusing in on a region containing the *Immune Infiltrate* and *Productive T cell & Tumor* CNs, the fluorescence data confirmed that this is an area of tumor inflammation (PDL1^+^, red; H2Kb^+^, yellow) and immune infiltration (CD45^+^, green) (Figure 5E). At the single-cell level, in *Productive T cell & Tumor* CNs (blue, Figure 5F) fluorescent images show CD8^+^ T cells (white membranes and white arrows, Figure 5G) are in contact with tumor cells. Also, these are abutted by CD8^+^ T cells that are next to stromal and immune cells (CD45^+^, green, Figure 5G) in *Immune Infiltrate* CNs (light blue, Figure 5F).

In tumors given canonically activated *T cells*, there was a shift to a higher proportion of the *Unproductive T cell & Tumor* CN (Figure 5H). Particularly the edge along the *Immune Infiltrate* and *Productive T cell & Tumor* CNs represents an important interface devoid of *Unproductive T cell Tumor* CNs. There was nearly a 4-fold increase in frequency of cells in this category in tumors given *2HC T cells* relative to those given *T cells* (Figure 5I, p < 0.05). This indicates that the *Unproductive T cell & Tumor* CN disrupts the *Immune Infiltrate* and *Productive T cell & Tumor* interface in tumors treated with *T cells* than tumors treated with *2HC T cells*.

Furthermore, the intersection between the *Immune Infiltrate* (light blue) and *Productive T cell & Tumor* (blue) CNs was greater in tumors given *2HC T cells* than in those given *T cells* (Figure 5J). In contrast, the intersection of the *Unproductive T cell & Tumor* with both *Immune Infiltrate* and *Productive T cell & Tumor* CNs was greatest in the tumors treated with *T cells* (Figure 5J). Thus, the phenotype of the therapeutic T cell influences its capacity to shape coordinated immune infiltration that support productive T cell tumor-killing zones.

### A subset of therapeutic T cells express anti-inflammatory cytokines that are associated with greater levels of regulatory environments

**Figure 6:**
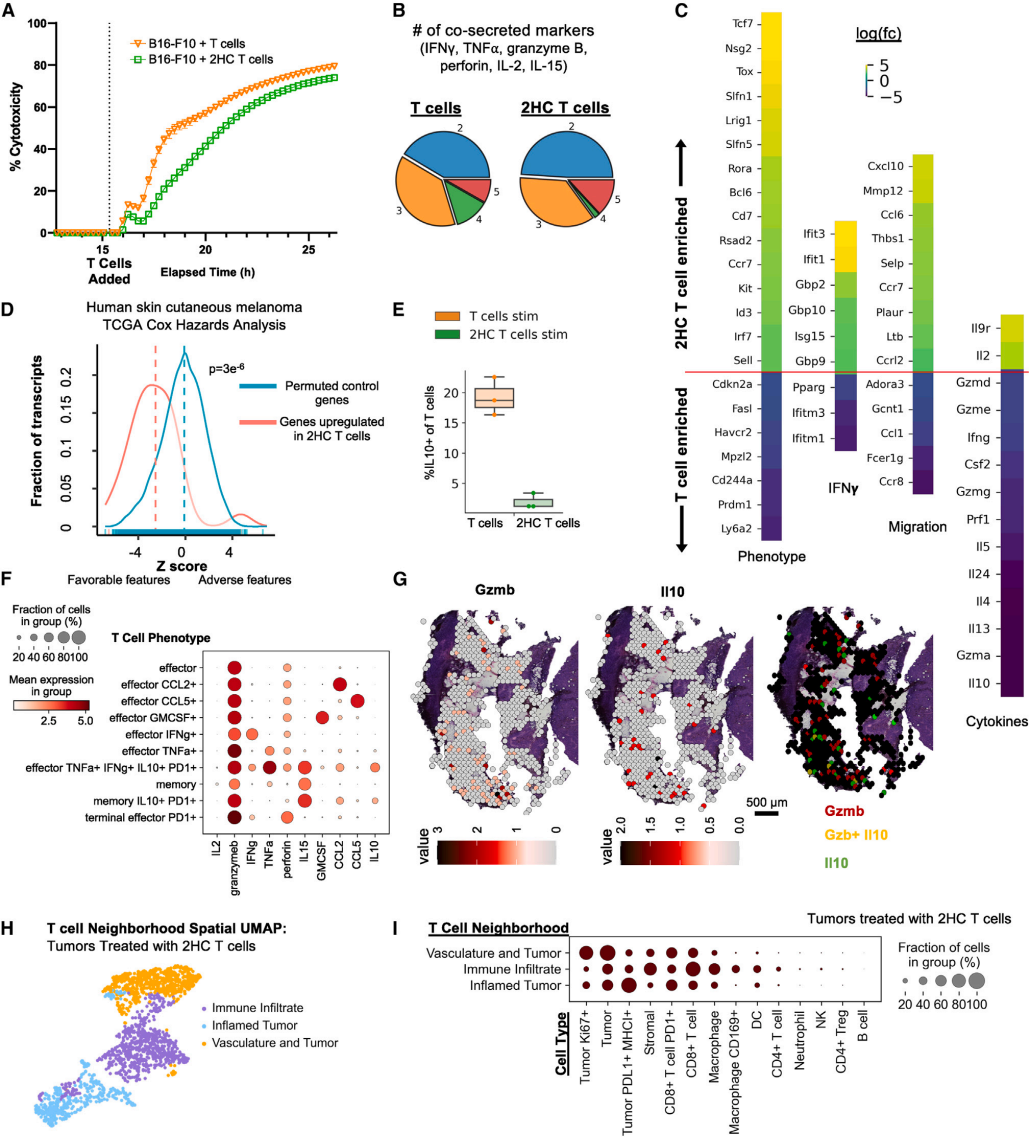
A fraction of T cells activated under standard conditions express anti-inflammatory cytokines at levels greater than metabolically treated T cells (A) Percent cytotoxicity in an *in vitro* killing assay (n = 3 replicates, representative of two independent experiments). (B) Pie charts of polyfunctionality of T cells (averages n = 4 replicates). (C) log2 fold differences in gene expression. (D) A density plot showing the distribution of multivariate Cox model gene expression *Z* scores for the 2HC T cell-enriched gene set. (E) Proportion of IL-10^+^ cells as determined by CyTOF analysis (n = 3 replicates, p < 0.05 two-sided t test). The boxplots show the median (center line), 25th to 75th percentile (box limits), minimum and maximum values (whiskers), and outliers (points outside 1.5× the interquartile range). (F) Individual cell phenotypes characterized by CyTOF phenotyping (n = 3 replicates). (G) Spatial transcriptomics expression of GZMB, IL-10, or both in tumor from mouse treated with T cells (without 2HC) and harvested on day 3. (H) Spatial UMAP plots of T cell neighborhood composition vectors across all experiments with T cells from tumors treated with 2HC-modified T cells. (I) T cell neighborhood cell-type composition per individual T cell neighborhoods.

Next, we looked into why 2HC T cells may cause more compartmentalized productive tumor interfaces. Interestingly, T cells activated with or without 2HC kill tumor cells with the same efficiency (Figure 6A). Furthermore, similar levels of effector molecules and similar levels of polyfunctionality^38^^,^^39^ (i.e., percentages of cells able to produce multiple effector molecules at once) were observed for T cells activated with and without 2HC (Figures 6B and S9A).

RNA-sequencing (RNA-seq) data indicated that the expression of genes enriched in *2HC T cells* over *T cells* was also found to be associated with favorable disease outcomes (represented by lower *Z* scores) relative to randomly permuted gene sets of the same size within human skin cutaneous melanoma samples from The Cancer Genome Atlas (TCGA) (Figures 6C and 6D). Further analysis with gene ontology enrichment analysis showed enrichment for pathways relating to immune cell differentiation, interferon signaling, and cytokines (Figure S9B, Extended data). Consequently, we compared the two T cell treatment conditions with each other highlighting the greatest log-fold change expression of genes falling into these major pathways: phenotypic, interferon (IFN)γ signaling, migration, and cytokine-related genes (Figure 6C).

Differences in phenotypic genes agreed with the CyTOF phenotypic characterization (Figure S5 and 6C). Moreover, in a separate study, we showed that IFNγ plays a role in transformation of tumor phenotype.^34^ Particularly interesting to tumor cellular organization, *CXCL10* was enriched within *2HC T cells* (Figure 6C), known to be a T cell recruitment factor.^40^ In the CODEX data, we observed higher CD8^+^ T cell density in tumors of both treated groups that was most apparent in tumors treated with *2HC T cells* (Figure S9C). *LTB* is also enriched within *2HC T cells* (Figure 6C) and is involved in lymphangiogenesis, which aligns with our observation of 2-fold increased levels of lymphatic cells in tumors from mice given *2HC T cells* (Figure S5H).

Interestingly, *T cells* also had higher levels of expression of mRNAs encoding anti-inflammatory cytokines *IL5*, *IL4*, *IL10*, and *IL13* (Figure 6C). The expression of anti-inflammatory cytokines may explain the higher levels of the regulatory neighborhood *Unproductive T cell & Tumor* within tumors given *T cells*. However, RNA-seq data provide a population-level average of expression, and we observed diverse populations in the single-cell CyTOF data (Figures 3A and 3B). Consequently, we confirmed with CyTOF that a greater percentage of *T cells* secrete IL-10 than do *2HC T cells* (∼19% versus 1.5%) (Figure 6E, p < 0.001). Interestingly, the cell types that expressed IL-10 also expressed other effector molecules and PD1 (Figure 6F). We confirmed this using spatial transcriptomics, where we observe 12.5% of the GZMB+ spots were positive for IL-10 (Figure 6G, panels 2 and 3). This supports the idea that a fraction of T cells that secrete anti-inflammatory molecules may counteract development of effective T cell tumor-killing microenvironments that are supported by immune cells. Taken together, these *in vivo* and *in vitro* data indicate that T cell phenotypes, not related to tumor-killing efficacy or traditional effector molecules, influence the ability of T cells to curate productive tumor-killing microenvironments.

Moreover, TCNs of *2HC T cells* indicated the *immune infiltrate* neighborhood also shares proximity in the UMAP space with the *inflamed tumor* TCN (Figures 6H and 6I). This finding agrees with our analyses that indicate the importance of the border of a dense immune infiltration TCN and T cell killing zones with inflamed tumor cells (Figures 4 and 5). Accordingly, this indicates that 2HC T cells curate the tumor microenvironment and enable broad immune cell infiltration that supports an effective anti-tumor T cell killing zone.

### Coordinated interactions of CNs orchestrate anti-tumor immune responses in patients with metastatic melanoma receiving immune-checkpoint inhibitors

**Figure 7:**
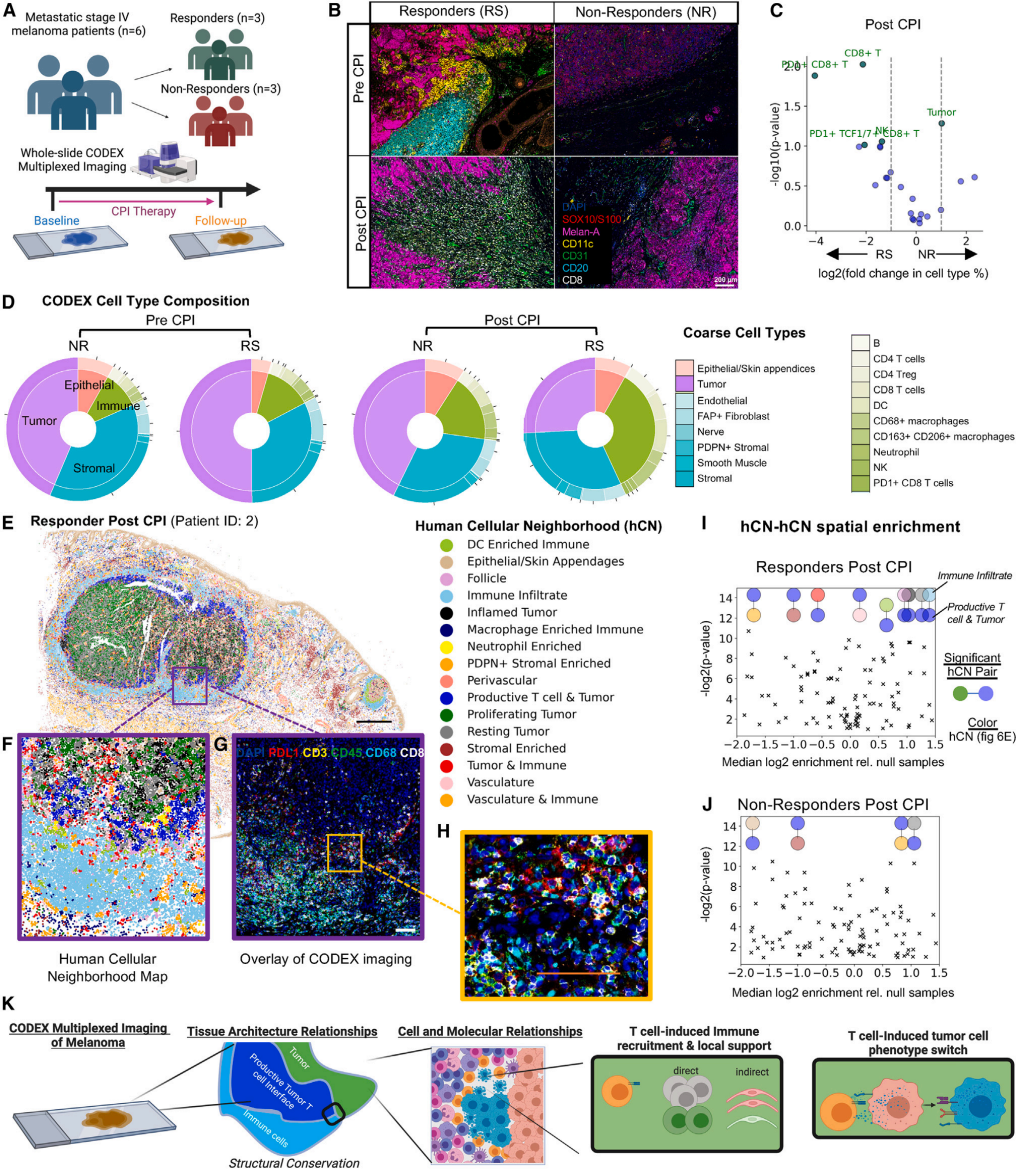
CODEX analysis of pretreatment and post-treatment samples from metastatic melanoma patients who received immune-checkpoint inhibitor therapy reveals distinct changes in T cell infiltration characterizing responding patients similar to changes observed for murine B16F10 experiments (A) Schematic representation of the study design. (B) Representative CODEX multiplexed imaging overlays (6/58 markers shown) for responders and non-responders, pre and post therapy (scale bar, 200 μm). (C) Volcano plot of the log2 of the fold change in cell-type percentage of non-responders (NR) to responders (RS). (D) Donut charts representing overall major cell-type compartments from CODEX multiplexed imaging (inside donut), and coarse grain cell-type percentages (outside donut, with legend) for all four groups shown. (E) Human cellular neighborhood (hCN) map from one of the patient’s tumor samples that was imaged by CODEX multiplexed imaging taken post-checkpoint inhibitor (CPI) therapy (black scale bar, 1 mm). (F) Magnified portion of the human cellular neighborhood (hCN) map indicating a location where the *Immune Infiltrate* hCN (sky blue) is adjacent to the *Productive T cell & Tumor* hCN (blue). (G) The same area magnified but showing a seven-color overlay (out of 58 total markers) highlighting immune cell infiltration (scale bar, 100 μm) next to areas where (H) CD8^+^ T cells are engaging with tumor cells with an inflamed phenotype (orange scale bar, 100 μm). (I and J) Significant two-combination neighborhood motifs enriched or depleted that are found associated with the *Productive T cell & Tumor* hCN compared with null distribution of permuted samples for (I) tumors from responders and (J) post therapy. Color represents shared neighborhood found across all samples and corresponding colors can be found in (E).

To see if there is similar coordinated CN behavior within effective immune responses to solid tumors of human patients, we collected tumor samples from patients with metastatic stage IV melanoma before and after treatment with immune-checkpoint inhibitors (CPIs) (Figure 7A). We then performed CODEX multiplexed imaging on the 12 whole-slide sections with a panel of 58 antibodies targeting major immune, stromal, and tumor compartments. Even from gross qualitative examination of the CODEX imaging we observed substantially higher degree of inflammation within responder (RS) tumors, particularly post CPI (Figure 7B).

While we observed no significant differences in the abundance of major cell types between RS and NR at baseline (Figures S10A and S10B), our results show that greater abundance of PD-1^+^ CD8 T cells and TCF1/7^+^ CD8 T cells was associated with patients responding to treatment (Figures 7C, S10C, and S10D). Indeed, post-CPI therapy, responders showed a strong increase in the fraction of immune cells at the tumor (Figure 7D). This major increase in the immune cell compartment was particularly driven by a strong influx of CD8^+^ T cells, NK cells, DCs, and macrophages, and a decrease in tumor cells when comparing pretreatment with post-treatment samples of RS as compared with NR tumors (Figures 7C and S10D).

We found 16 unique human melanoma cellular neighborhoods (hCNs) (Figures 7E and S11A), of which many were compositionally similar to the neighborhoods we previously observed in our mouse melanoma tumor samples, though we also observed some new tissue structures (Figure S11A). Two of the hCNs that were compositionally similar to murine tumor CNs were the *Immune Infiltrate* hCN and the *Productive T cell & Tumor* hCN *(*Figure S11A). Akin to what we observed in the murine tumors, we see shared proximity of the *Immune Infiltrate* hCN with the *Productive T cell & Tumor* hCN at the tumor interface (Figure 7E–H).

Pre-CPI therapy, the *Productive T cell & Tumor* hCN was largely associated independent or with major tumor hCNs (Figure S11B). However, post CPI, the *Productive T cell & Tumor* hCN was almost exclusively found co-associated with the *Immune Infiltrate* hCN (Figure S11C). Permutation testing of hCN instances also identified this shift in organization of the *Productive T cell & Tumor* hCN within responders after CPI therapy, including highly enriched colocalization with the *Immune Infiltrate* hCN (Figure 7I). This hCN-hCN spatial colocalization was not observed pre-CPI (Figure S11D). This contrasts with the *Productive T cell & Tumor* hCN in post-CPI samples for NR, where there was no spatial co-enrichment with the *Immune Infiltrate* hCN at either timepoint (Figures 7J and S11E).

These results corroborate our earlier findings within murine melanoma tumors and suggest that an active and robust anti-tumor CD8^+^ T cell response can cause and is dependent on neighboring immune-dense microenvironments. This is also evident from the fact that the *Productive T cell & Tumor* hCN does not increase in frequency pre to post CPI, though the *Immune Infiltrate* hCN does (Figure S11F), indicating recruitment upon a more robust CD8^+^ T cell response. Similarly, the overall tissue size of *Immune Infiltrate* hCN instances (hCNs of the same type spatially connected) increased post CPI in responders. This result, in addition to the fact that we observe globally larger hCN instances from responders compared with non-responders post CPI (Figures S12A and S12B), indicate a less fragmented immune organization within the tumor microenvironment. Taken together, these data suggest that organized immune infiltration areas abutting robust anti-tumor T cell zones is necessary for prolonged and effective anti-tumor efficacy.

## Discussion

In this study, we characterized the spatial cellular microenvironments that anti-cancer T cells are found in over time and across T cell phenotypes. Critically, the studies demonstrate the ability, by simple empiric data extraction, to derive an inference history of immune cell infiltration of a tumor along with mechanistic insights into cell-cell interactions that are critical for T cell-mediated progression toward tumor eradication.

We leveraged multiplexed imaging techniques to explore the cellular microenvironments within B16F10 tumors treated with activated cancer-specific T cells. Multiplexed imaging demonstrated that T cell therapies drive tumor restructuring and lead to changes in tumor cell phenotypes. Higher levels of tumor cells with the inflamed phenotype were correlated with increased levels and proximities of CD8^+^ T cells (Figures 7K and S12C). In parallel work, multiscale modeling also indicated that therapeutic T cells convert tumor cells to an inflamed, anti-proliferative phenotype at scales larger than direct cell-cell interaction critical for tumor control.^34^ The application of T cell therapies that allow for the targeted remodeling of tumor microenvironments might therefore help to overcome primary or acquired resistance to existing treatments for advanced melanoma, such as checkpoint-blocking antibodies.

In addition to changing the tumor phenotype, we demonstrate that T cells can mediate the formation of either productive or unproductive tumor T cell neighborhoods that are co-enriched with inflamed tumor cells. Metabolic manipulation of T cells,^11^ co-induced broad immune infiltration that shared borders with productive T cell and tumor neighborhoods. Further, the immune infiltrate and productive T cell and tumor neighborhoods were not broken up by regulatory neighborhoods as they were in tumors of mice treated with T cells activated using the standard protocol. We similarly observed only in patients responding to CPI therapy a pronounced interaction between the productive T cell and tumor cellular neighborhood and the immune infiltrate neighborhood in samples post-CPI start. Particularly, these CNs, similar to those found in murine tumors, also had greater compartmentalization and were characterized by a higher abundance of T cells with a memory phenotype as opposed to non-responding patients. This further affirms previous findings that dense immune infiltrates as opposed to diffuse or unorganized immune infiltrates are beneficial for tumor outcomes^33^^,^^41^^,^^42^ and adds to how *in situ* cellular relationships provide spatial mechanisms of homeostasis and disease.^21^^,^^43^^,^^44^^,^^45^^,^^46^^,^^47^^,^^48^^,^^49^^,^^50^^,^^51^^,^^52^^,^^53^

Next, our findings that the T cell phenotype influences structural reprogramming of the tumor microenvironment adds to the list of factors known to be critical for determining therapeutic T cell success, such as effector molecule expression and T cell longevity. Here we saw that canonically activated T cells have subset populations that secrete anti-inflammatory cytokines in addition to inflammatory molecules. We found that therapeutic T cells express IL-10. IL-10 is known to be expressed by Tregs,^54^ regulate CD8^+^ T cell responses,^55^ inhibit activity of myeloid cells,^56^ and be implicated in relapse of CAR T therapy in acute lymphoblastic leukemia.^57^ The presence of these T cell phenotypes may explain why regulatory microenvironments disrupt productive T cell and tumor responses more in tumors treated with these T cells than with metabolically activated T cells. Our study supports the idea that T cell therapies should be designed with cytokine and chemokine expression in mind. This will require new methods of evaluating *ex vivo* T cell phenotypes to include molecules that are involved in tissue restructuring and, notably, anti-inflammatory processes.

One new spatial analysis we developed was a method of collapsing a cell’s microenvironment into lower dimensional space. We used this to evaluate similarity of composition of CNs, similarity of neighborhoods across different conditions, and connections back to temporal sequences. Particularly, we leveraged this new technique to define T cell-specific CNs within the tumor across time and treatment. The T cell-specific neighborhoods represented major changes necessary within the microenvironment for the anti-tumor response: tumor infiltration from vasculature, immune cell recruitment (particularly changes in antigen-presenting cells with influx of dendritic cells and macrophages), and induction of tumor death and inflammation. Here we were able to characterize differences in the microenvironments of adoptively transferred T cells compared with endogenously recruited T cells.

In summary, our results demonstrate that therapeutic T cells shape the tumor cellular microenvironment and depend on the input of the therapeutic T cell phenotype. Moreover, our results are the first to our knowledge to investigate the temporal dynamics of immune responses in the course of tumor progression and relationships of higher-order spatial biology that are correlated with success of the therapy. In this regard, our results stress the importance of compartmentalized immune infiltration bordering the edge of T cell attack of tumor cells. Thus, this dataset is an archetype for understanding and evaluating cell-cell interaction *in situ* for cellular therapies. It calls for transformative changes to how T cell therapies should be screened prior to transfer and suggests additional avenues for engineering of T cell therapies to prioritize cellular microenvironment manipulation to yield more productive anti-tumor immune responses.

### Limitations of the study

Here we used an established, syngeneic murine model of melanoma that allowed analysis of the native immune response, therapeutic perturbations, and dynamic restructuring. This work and the findings in this model can be followed up in additional mouse tumor models of both CAR T and endogenous T cells, along with additional perturbations. Our multiplexed imaging panel only included 42 antibodies for mouse and 58 antibodies for human, which may not fully represent all cell types or states that are meaningful in the tumor, and our panels could be expanded by developments in multiplexed imaging technologies such as single-cell spatial transcriptomics.^44^^,^^58^^,^^59^ Here, we characterized very large areas of the tumor from a limited set of human melanoma patients (six total from two timepoints), and future studies incorporating more patients through smaller tissue microarrays could be studied using multiplexed imaging.

## STAR★Methods

### Key resources table

**Table:** 

REAGENT or RESOURCE	SOURCE	IDENTIFIER
**Antibodies**		

Custom-conjugated CyTOF antibodies	Table S2.xlsx	All information on clones, companies, RRIDs, etc. are included within these detailed tables
Custom-conjugated human CODEX antibodies	Table S2.xlsx	
Custom-conjugated murine CODEX antibodies	Table S2.xlsx	
anti-CD3 (clone 145-2C11)	Bioxcell	Catalog #BE0001-1; RRID: AB_1817016
anti-CD28 (clone 37.51)	Bioxcell	Catalog #BE0015-1; RRID: AB_1817016
TruStain FcX™ (anti-mouse CD16/32) Antibody	Biolegend	Catalog #101319; RRID: AB_2783137

**Biological samples**		

Human FFPE melanoma blocks	University Medical Center Mainz	See Table S1 for details

**Chemicals, peptides, and recombinant proteins**		

Potassium hydroxycitrate tribasic monohydrate	Sigma-Aldrich	59847-1G
gp100 - KVPRNQDWL	AnaSpec	AS-62589
Recombinant Human IL-2 (carrier-free)	Biolegend	589106
Protein Transport Inhibitor (Containing Monensin)	BD Biosciences	554724
Protein Transport Inhibitor (Containing Brefeldin A)	BD Biosciences	555029
Recombinant Mouse IFN-γ (carrier-free)	Biolegend	575302
cis-Diammineplatinum(II) dichloride (cisplatin)	Sigma-Aldrich	P4394-25MG
EMS 16% Paraformaldehyde aqueous	Fisher	50-980-487

**Critical commercial assays**		

xCELLigence Real-Time Cell Analysis	Agilent	Single Plate
PureLink RNA Mini Kit	ThermoFisher	12183018A
NEBNext Poly(A) mRNA magnetic Isolation Module	New England Biolabs	E7490
NEBNext Ultra II Directional RNA Library Prep Kit for Illumina	New England Biolabs	E7760
NEBNext Multiplex Oligos for Illumina	New England Biolabs	E7500
Visium Tissue Optimization slides	10x Genomics	3000394
Visium Spatial Gene Expression slides	10x Genomics	2000233

**Deposited data**		

All single cell deposited data	Dryad	https://doi.org/10.5061/dryad.k0p2ngfcc

**Experimental models: Cell lines**		

B16-F10 tumor cell line	ATCC	RRID:CVCL_0159

**Experimental models: Organisms/strains**		

B6 - C57BL/6J	Jackson Laboratories	RRID:IMSR_JAX:000664
PMEL - B6.Cg-Thy1a/Cy Tg(TcraTcrb)8Rest/J	Jackson Laboratories	RRID:IMSR_JAX:005023

**Software and algorithms**		

CODEX Processor	https://github.com/nolanlab/CODEX	
Segmenter	https://michaellee1.github.io/CellSegSite/index.html	
Neighborhood analysis	https://github.com/nolanlab/NeighborhoodCoordination	
Tissue Schematic Analysis	https://github.com/nolanlab/TissueSchematics	
Scanpy	https://scanpy.readthedocs.io/en/stable/	
Galaxy	https://usegalaxy.org/	
ImageJ	https://imagej.net/software/fiji/	
STutility	https://ludvigla.github.io/STUtility_web_site/	

**Other**		

Fisherbrand™ Superfrost™ Plus Microscope Slides	Thermo Fisher Scientific	12-550-15

### Resource availability

#### Lead contact

Further information and requests for resources and reagents should be directed to and will be fulfilled by the lead contact, Garry Nolan (gnolan@stanford.edu).

#### Materials availability

No shareable materials were created in this manuscript.

#### Data and code availability

• We also provide processed, quantified and annotated single-cell CODEX datasets with labeled cell types, neighborhoods, and also protein expression at Dryad for the time course datasets, different T cell phenotype studies, and the human data. It is available for download from dryad: https://doi.org/10.5061/dryad.k0p2ngfcc.
• This paper does not report original code
• Any additional information required to reanalyze the data reported in this work paper is available from the lead contact upon request

### Experimental model and study participant details

#### Cell-lines

B16-F10 melanoma cells were obtained from ATCC catalog CRL-6475 and maintained at low passage number.

#### Mice

B6 and PMEL transgenic mice were maintained as per guidelines approved by Stanford University’s Laboratory Animal Care (APLAC) Institutional Review Board (Protocol number 33502). All experiments conform to the relevant regulatory standards. C57BL/6J and PMEL mice were purchased from Jackson Laboratories.

#### Human samples

A cohort of 50 patients who were diagnosed with metastatic stage IV melanoma and who received first-line immune-checkpoint inhibitor therapy with either nivolumab, pembrolizumab or ipilimumab plus nivolumab between 2015 and 2019 at the University Medical Center Mainz, Germany were identified for this study. Among this cohort, 6 patients with survival follow-up data until April 2023 were retrospectively identified according to the following selection criteria (see also Figure 7A): Histopathological confirmed diagnosis of melanoma, sufficient pre and post-checkpoint inhibitor therapy start tissue available, >30% uncompromised tumor tissue, complete follow-up with documentation of treatment outcomes including best overall response according to RECIST criteria, progression-free survival and overall survival (for details see Table S1). Details on clinical-pathological parameters at initial diagnosis were collected from the patient's medical records.

The studies involving human participants were reviewed and approved by the local ethics committee of the University Medical Center Mainz (Ethik-Komission der Landesärztekammer Rheinland-Pfalz, No: 2020–14822). The patients provided written informed consent to participate in the study. The analysis of the anonymized retrospective data was conducted according to the guidelines of the Declaration of Helsinki.

### Method details

#### T cell culture and stimulation

##### Immune cell isolation

Murine cells were obtained from adult mouse lymph nodes and spleens. Obtained cells were treated with ACK lysis buffer to lyse red blood cells, and lysates were filtered through cell strainers to isolate splenocytes.

##### T cell media

The T cell media was RPMI 1640 supplemented with glutamine, 1x non-essential amino acids, 1 mM sodium pyruvate, 0.4x vitamin solution, 92 μM 2-mercaptoethanol, 10 μM ciprofloxacin, and 10% fetal bovine serum.

##### CD3-coated plate preparation

To each well of a 96-well, U-bottomed plate we added 50 μL of a solution of 5 μg/mL anti-CD3 (Bioxcell, clone 145-2C11) in PBS. After incubation at 4°C overnight, liquid was decanted.

##### T cell stimulation

Isolated murine immune cells (all splenocytes harvested) were stimulated by incubation with 1 μM cognate peptide gp100 (KVPRNQDWL) and 50 IU/mL IL-2. Cells were seeded at a density of 2-5×10^6^ cells/mL. Cells were fed with additional IL-2 in T cell media every other day. On day 5, cells were added to CD3-coated plates in culture media containing 2 μg/mL anti-CD28 (Bioxcell, clone 37.51). On day 8 cells were removed from plates and plated on uncoated plates and fed with IL-2-containing media until day 10.

##### 2HC T cell stimulation

For stimulation in the presence of 2HC, we followed the protocol for T cell stimulation as described above. The only exception was that 2HC was prepared sterile within T cell media and then added to culture media at a concentration of 5 mM and maintained within culture every time cells were fed or exchanged with media on days 3, 5, 8.

#### *In vitro* T cell killing assay

T cell killing and tumor cell growth rates were determined using the xCELLigence Real-Time Cell Analysis platform. Wells of xCELLigence E-plates were coated with gold nanoparticles, and electrical potential was passed across the plate every 15 min. Monitoring of changes in the electrical impedance enabled quantification of adherent cells over time. For these assays, T cells were expanded. B16-F10 melanoma cells were split and then left as control cells or treated with 10 ng/mL IFNγ for 24 h prior to plating. After the pretreatment, 10,000 B16-F10 cells were plated in each well of the xCELLigence E-plate and allowed to adhere for 12 h. Next, T cells were added at 8:1 effector to target ratio. The growth of tumor cells and killing by T cells was monitored for up to 24 h. Killing was calculated by normalizing the cell index of each well to the time point just before addition of the T cells and then quantifying the differences between T cell and control wells without T cells over time.

#### CyTOF phenotyping

##### Antibodies

Primary antibody transition metal-conjugates were prepared in-house using 100-μg antibody lots and the MaxPAR antibody conjugation kit (DVS Sciences) according to the manufacturer’s recommended protocol. Following conjugation, antibodies were diluted in Candor PBS Antibody Stabilization solution and stored at 4°C. Information on CyTOF staining panels (phenotyping, ICS, and IL-10 panels) is given in Table S2.

##### Surface staining

Following T cell stimulation on day 10, 2×10^6^ cells were stained by addition of 1 mL of 25 μM cisplatin in PBS. After 1 min at 4°C, the reaction was quenched with 1 mL of fetal bovine serum, and cells were washed with cell staining medium (CSM; PBS with 0.5% bovine serum albumin and 0.02% sodium azide). Cells were blocked with FcBlock (0.25 μg/1×10^6^ cells) for 15 min at room temperature, then the surface antibody cocktail was added and incubated 1 h at room temperature on a shaker at 100 rpm. Cells were washed with CSM and then with PBS. Cells were fixed and stained with intercalators overnight at 4°C in a solution of 1.6% paraformaldehyde in PBS. The next day, the cells were washed once with CSM and twice with doubly distilled water, resuspended in doubly distilled water, and analyzed using CyTOF.

##### ICS/IL10 staining

Following T cell stimulation on day 10, 2×10^6^ cells were isolated from each condition and separated into restimulation and no stimulation groups in 100 μL T cell media at 500,000 cells per well in a 96 u-bottom plate. Samples were added to previously coated anti-CD3 plates, and anti-CD28 was added to the culture media at 2 μg/mL. To inhibit protein transport, 1:350 BD GolgiStop Protein Transport Inhibitor (BD Biosciences), and 1:350 BD GolgiPlug Protein Transport Inhibitor (BD Biosciences) in T cell media was added to the samples. Both groups were incubated at 37°C for 4–6 h. Cells were washed with PBS then stained with cisplatin at 25 μM in PBS in 1 mL for 1 min at 4°C, quenched with 1 mL of fetal bovine serum, and washed with CSM. Cells were blocked with FcBlock (0.25 μg/1×10^6^ cells) for 15 min at room temperature, then the surface antibody cocktail was added and incubated 1 h at room temperature on a shaker at 100 rpm. Cells were washed with CSM and then with PBS. Then cells were then fixed and permeabilized with 500 μL BD Cytofix/Cytoperm Fixation and Permeabilization Solution (BD Biosciences) 20 min at room temperature or 4°C overnight. Cells were then washed with 1× BD PERM/Wash buffer with 2% bovine serum albumin twice. Cells were stained with intracellular cytokine antibodies in PERM/Wash buffer with 2% bovine serum albumin at room temperature for 45 min, washed again in PERM/Wash buffer with 2% bovine serum albumin, and then washed with PBS. Cells were then fixed and stained with intercalators overnight at 4°C or 20 min at room temperature in a solution of 1.6% paraformaldehyde in PBS. The next day, the cells were washed once with CSM and twice with doubly distilled water, resuspended in doubly distilled water, and analyzed using CyTOF.

#### Mouse tumor model

On day 0, B6 mice were injected with 2 × 10^5^ B16-F10 melanoma tumor cells. On day 0, immune cells were isolated from a PMEL mouse and stimulated as described above with or without 2HC for 10 days to produce stimulated T cells for adoptive transfer. On day 9, mice were given a central dose of 500 cGy, which induces transient lymphopenia.^60^ On day 10, T cells cultured *ex vivo* were harvested and adoptively transferred intravenously in volumes of 100 μL with 1×10^6^ of conventionally stimulated or 2HC-activated T cells per mouse. Tumors were harvested 1, 3, 5, or 12 days after adoptive transfer.

#### CODEX multiplexed imaging

##### Array creation

Imaging data was collected from multiple mice from multiple experiments. We included up to six tumors into a single array, which were subsequently cut onto the same coverslip. Arrays were constructed on the cryostat and sectioned at a width of 7 μm. Available human tumor tissue was cut with a 3 μm section thickness and mounted onto Superfrost PLUS slides for further processing. For human tumors, we investigated a total of 12 tissues (each 6 pre-treatment and 6 post-treatment) from 6 metastatic melanoma patients that were treated with immune-checkpoint inhibitors all placed on individual slides.

##### CODEX antibody conjugation and panel creation

CODEX multiplexed imaging was executed according to the CODEX staining and imaging protocol.^29^ CODEX imaging involves iteratively annealing and stripping of fluorophore-labeled oligonucleotide barcodes complimentary to the barcodes attached to 40+ antibodies used to stain the tissue. Antibody panels were chosen to include targets that identify subtypes of tumor, stromal, innate, and adaptive immune cells. Detailed panel information can be found in Table S2. Each antibody was conjugated to a unique oligonucleotide barcode, after which the tissues were stained with the antibody-oligonucleotide conjugates. We validated that staining patterns matched patterns observed by immunohistochemical analysis within positive control tissues of tumor or mouse spleen. Antibody-oligonucleotide conjugates were first tested and titrated in low-plex fluorescence assays, and signal-to-noise ratio was evaluated, then antibody-oligonucleotide conjugates were tested together in a single CODEX multicycle. Signal-to-noise ratio was again evaluated, and the optimal dilution, exposure time, and appropriate imagine cycle was determined for each conjugate (Table S2).

##### CODEX multiplexed imaging

The tissue arrays were stained with the validated panels of CODEX antibodies and imaged.^29^ Briefly, this entailed cyclic stripping, annealing, and imaging of fluorescently labeled oligonucleotides complementary to the oligonucleotide conjugated to the antibody. Each array underwent CODEX multiplexed imaging; metadata from each CODEX run can be found in Table S2.

#### RNA-seq

##### Library preparation

RNA isolation was performed with PureLink RNA Mini Kit (ThermoFisher, USA) following the protocol for monolayer cells. The NEBNext Poly(A) mRNA magnetic Isolation Module (E7490, New England Biolabs) was used for mRNA purification. For the library preparation, the NEBNext Ultra II Directional RNA Library Prep Kit for Illumina (E7760) and NEBNext Multiplex Oligos for Illumina (E7500) were used. The quality of the library was checked using an Agilent Bioanalyzer. RNA-seq libraries were sequenced by the Stanford Genomics Core service, The sequencing data was generated with instrumentation purchased with NIH funds.

#### Spatial transcriptomics

##### Visium spatial gene expression acquisition

B16-F10 tumors were embedded in OCT (TissueTek Sakura) and cryo sectioned at 10-μm thickness at −20°C. Tissue sections were placed on Visium Tissue Optimization slides (3000394, 10x Genomics) and Visium Spatial Gene Expression slides (2000233, 10x Genomics). The tissue optimization sample slide and spatial gene expression slide were processed according to the manufacturer’s protocols.

### Quantification and statistical analysis

#### CODEX multiplexed imaging data analysis

##### CODEX data processing

Raw imaging data were processed using the CODEX Uploader for image stitching, drift compensation, deconvolution, and cycle concatenation. CODEX enables single-cell resolution protein quantification that can be used for evaluating cell type definition, state, and location. To obtain quantitative single cell information, we processed the multiplexed imaging data, segmented individual cells, and extracted single-cell protein expression. Processed data were then segmented using the CellVisionSegmenter, a neural network R-CNN-based single-cell segmentation algorithm.^61^ Both the CODEX Uploader and Segmenter software can be downloaded from our GitHub site (https://github.com/nolanlab/CODEX), and the CellVisionSegmenter software can be downloaded at https://github.com/michaellee1/CellSeg. After the upload, images were evaluated for specific signal: Any markers that produced an untenable pattern or a low signal-to-noise ratio were excluded from the ensuing analysis. Uploaded images were visualized in ImageJ (https://imagej.nih.gov/ij/).

##### Cell-type analysis

A total of 1,052,125 cells were identified and classified into 23 cell types and states based on marker expression for murine studies. We segmented 5,019,159 individual cells from the 12 CODEX human images and identified 39 major cell types based on their expression profiles. Cell type identification were done following the strategies we have developed.^29^^,^^52^ Briefly, nucleated cells were selected by gating DRAQ5, Hoechst double-positive cells, followed by z-normalization of protein markers used for clustering (some phenotypic markers were not used in the unsupervised clustering). The data were overclustered with Leiden-based clustering with the scanpy Python package. Clusters were assigned a cell type based on average cluster protein expression and location within image. Impure clusters were split or reclustered following mapping back to original fluorescent images.

##### Cell type across multiple tissue slices

We imaged with CODEX multiplexed imaging 2-3 sections separated by ∼1 mm for the same tumor from each mouse. More variation is observed between mouse-to-mouse replicates within an experiment, and the most variation is observed between experiment replicate to experiment replicate.

##### Neighborhood identification analysis

Neighborhood analysis was performed based on code and settings we have developed.^33^ Briefly a window size of 10 nearest neighbors was taken across the tissue cell-type maps. Cells of each type were quantified for each of these windows. We correlated the abundance of the neighboring cell types to each other for all cell types and identified distinct modules of cells that co-exist within the tumor microenvironment. Also, these vectors were clustered into commonly composed neighborhoods. We both employed k-means elbow plot and overclustering to inform our choice for the number of clusters of cellular neighborhoods. For overclustering, we would generate 20–30 clusters and map the clusters back to the tissue and evaluate for cell-type enrichments to determine overall structure. Then with this evaluation we would merge down into the uniquely identified neighborhoods. CNs (e.g., Figure 2D, rows) were defined based on enrichment or depletion of certain cell types (e.g., Figure 2B, columns) as compared to average tissue percent composition.

##### Cell-type specific neighborhood identification analysis

For T cell-specific neighborhoods, we used the cell neighbor composition vectors by taking the 10-nearest neighbors surrounding the CD8^+^ T cells. We then clustered cell neighborhoods with k-means clustering. We also used these vectors for UMAP plots prepared using the scanpy package in Python. Thus, each point in the UMAP is a CD8^+^ T cell, and each relationship of points in the UMAP represents the similarity of one CD8^+^ T cell’s neighbor composition to another CD8^+^ T cell neighbor composition.

##### Cell interaction analysis

Cell interaction analysis was carried out using a method we have developed.^62^ Briefly, the Delaunay triangulation of cells were identified within each field of view using the x, y position. Cell to cell interactions within 35 μm from one another were identified and baseline distributions were established by performing the calculation for each iteration the cell label was randomly assigned existing x, y positions and compared to observed distances with a Wilcoxon Test. We used 1000 permutations for the distribution. The fold enrichment of distances between the observed data over the mean distances from the permutation test. The log fold of the distances for each cell type interaction where p values less than 0.05 were plotted for each group using ggplot2 in R. Selected pairs were chosen based on distance averages closer than expected for *2HC T cells* and difference in distance between *T cells*, except for the CD8^+^ T cell and Tumor PDL1^+^ MHCI^+^ combination.

Specifically, pairs were chosen that had a log fold of the distance less than −0.15 for *2HC T cell* condition and also a difference of 0.5 between the *2HC T cells* and *T cells* conditions.

##### Random forest analysis

This was conducted on cell type percentages using the randomforestSRC R package (version 2.3.0)^63^^,^^64^. During bootstrapping, a randomly chosen two-thirds of samples were used to train each tree and remaining (out-of-bag, OOB) samples were used for cross-validation and forest-related estimates. No training was conducted on OOB samples and no iterative parameter optimization was performed. Variables were then ranked by minimal depth (MD), a dimensionless statistic that measures variable predictiveness in tree-based models. MD is defined as the shortest distance between the root node of a tree and the parent node of a maximal subtree, which is the largest subtree whose root node splits on the variable. Smaller MD values indicate greater predictiveness and a tree-averaged threshold MD was used to classify variables as predictive.

##### Likelihood ratio test

Differential cell type or neighborhood percentage was done using a linear mixed-effects model via the R package lme4 1.1–27.1 to account for experimental replicate.^65^ Briefly, for each cell type or neighborhood type, the percentage was regressed with experimental replicate as a fixed effect. p values were estimated using a likelihood ratio test via the R package lmtest 0.9–38. Significance at a false discovery rate of 5% was estimated using the Bioconductor package qvalue (https://www.bioconductor.org/packages/release/bioc/html/qvalue.html), after confirmation that null p values followed an approximately uniform (0,1) distribution.

##### Spatial context maps

Spatial context maps were created using our code and settings we developed.^37^ Briefly, the spatial context analysis of CN-CN associations has some similarities with our method to identify CNs but also contains a few key differences. First, we used a larger window size (100 nearest neighbors) for each cell across the tissue. Second, we used CN labels instead of cell type labels to create the composition windows (Figure 4H). Third, instead of clustering the windows, we determined the combination that included the fewest CNs to make up more than 85% of the neighborhoods within that window (Figure 4H). This combination informs about prominent associations of CNs in the window, a feature we termed spatial context. Fourth, we counted each combination and connected the most prevalent combinations together into a spatial context map (Figure 4H).

To construct the graphs, the combination of the fewest neighborhoods that make up more than 85% of total percentage within that window are assigned to a specific combination. Next, the spatial contexts are quantified and plotted in a network graph structure with combination color representing the neighborhood combination and the size of the black circle indicating the relative proportion of the combination across the tissue. The row show the number of neighborhoods in combinations: row 1, a single neighborhood accounts for at least 85% of the neighborhoods surrounding the window; row 2, two neighborhoods make up more than 85% of the neighborhoods in the window; and row 3 and beyond, multiple neighborhoods are present in the window.

The number of CNs forming a combination provides insight into structural biological mechanisms. In some windows, a single CN made up more than 85% of all CNs. This indicates structural compartmentalization where there is similar cell type composition, and thus CN composition, compartmentalized across large areas of the tissue. In contrast to compartmentalization, spatial contexts with three or more CNs in a combination are indicative of CN mixing. The cases where a combination of two CNs make up at least 85% of a window are indicative of important interfaces and coordination of structures potentially necessary for function.

Spatial context analysis on all samples using shared CNs was used for Figure S9 that match the analysis done for Figure 4I from an individual experiment.

##### Barycentric plots for spatial context maps

First, all windows with any combination of *Immune Infiltrate* (light blue), *Productive T cell & Tumor* (dark blue), and *Unproductive T cell & Tumor* (red) neighborhoods that is greater than 85% were selected. Second, the relative frequencies of these neighborhoods were computed and then plotted in the barycentric coordinate system. To quantify this shift in T cell-tumor CN organization, we selected those cells with at least 95 of 100 neighboring cells in *Productive T cell & Tumor* and *Immune Infiltrate* CNs (Figure 5C, rectangle).

##### Instance segmentation

We measured overlap between neighborhood contacts, by constructing a tissue graph of the data.^37^ Briefly, a 10-nearest neighbor graph for the cells of each imaging region was constructed using the sklearn.nearest_neighbors module in Python. The connected components of this graph were identified using the scipy.sparse.csgraph module in Python. Instances that consisted of fewer than 5 cells were discarded. We calculated the total intersection of CN instances post identification, representing the shared border between each pair of the three CNs for all tumors.

##### Neighborhood-neighborhood motif identification

We used a similar method to instance segmentation to identify significantly associated cellular neighborhood-neighborhood motifs.^37^ Briefly, motif identification leverages computational identification of CN instances, or segmented areas of the tissue where multiple cells of the same CN are co-located instead of using individual cells. Then tissue network graphs are created that represent shared edges between instances of CNs and this is used to compute whether pairs of CNs are significantly associated more than a permuted null distribution.

To create a tissue graph for each treatment group, we took the union of the tissue graphs of each unique imaging region. We then created a null-set as the graph of the set of cell neighborhood assignments by a sequence of valid transpositions of cell neighborhood assignments. Permuting neighborhood assignments and fixing the number of vertices created the maximum entropy null distribution. Only two chains with at least five instances were considered. To identify significant chains, p values were Bonferroni corrected by multiplying by twice the number of tests conducted in each treatment group.

##### Canonical correlation analysis

Correlation analyses were performed using cell type compositions of neighborhoods.^33^ Briefly, for each cell neighborhood, the log cell neighborhood-specific cell type frequency of CD8^+^ T cells and CD8^+^ PD1^+^ T cells was computed. For each pair of cell neighborhoods, estimated canonical directions for the frequencies of these cells in each cell neighborhood was estimated using the scikit-learn Python package. For each pair of cell neighborhoods, replicates with no cells assigned to either neighborhood were not included in the analysis. The correlation of the projections along these canonical directions was compared to a permutation distribution, corresponding to 5000 random permutations of the data. The permutation p value (i.e., the percentage of permutations with an estimated canonical correlation the exceeded the observed one) was interpreted as the strength of communication.

#### RNA-seq data analysis

##### Primary data analysis

RNA-seq data was processed using Galaxy (https://usegalaxy.org/). First, *FASTQC* was run followed by the *Trim_galore* function that was done with a paired trim using other default settings. Next *RNA STAR* was run on the trimmed data using default settings with mouse reference m10 to align and eliminate unmapped reads/unannotated in bam files. Next the function *Run Feature Counts* was used and ran on paired runs. *Make data tabular* and *annotateMyIDs* was run using Entrez ID to generate read count per gene tables. Reads were normalized to count per million. Finally, genes were selected with expression of greater than 15 reads in either condition, and log2 fold change greater than 2.5 for gene ontology. Gene ontology analysis was done with the differentially enriched genes of 2HC T cells compared to T cells with http://geneontology.org/.

##### TCGA comparison analysis

To evaluate the prognostic significance of the gene set enriched in 2HC T cells, we used previously reported multivariate Cox proportional hazards model Z scores to assess the association of gene expression with survival.^66^ Briefly, this approach permits the use of gene expression measurements without dichotomization, uses right-censored survival data, and is adjusted for factors such as age and disease stage. We compared the Z scores for the gene set of interest to randomly permuted gene sets of the same size to determine p values.

##### Visium spatial data preprocessing

The standard preprocessing of the Visium spatial data was performed using the STutility package.^67^ Cells with more than 2000 reads and less than 10% mitochondrial gene expression were kept for downstream analysis. We harvested the tumors for these experiments 3 days after treatment. Since all T cells expressed granzyme B (Figure 6F), we used this marker to identify areas of T cells in the tumor (Figure 6G, panel 1).

## Supplemental information

Document S1. Figures S1–S12 and Table S1

Table S2. Supplementary information based on the CyTOF and CODEX panels within this paper (A) This tab has information about the CODEX antibody panel that was used to stain human melanoma samples. (B) This tab has information about the CyTOF antibody panels that were used within the study. (C) This tab has information about the CODEX antibody panel that was used to stain murine melanoma samples. (D) This tab has information about the CODEX antibody panel settings that were used to image CODEX-stained, murine melanoma samples. (E) This tab has information about the CODEX antibody panel settings that were used to image CODEX-stained, human melanoma samples.

Document S2. Article plus supplemental information
