# Supplementary material for: T cell-mediated curation and restructuring of tumor tissue coordinates an effective immune response
Source: Cell Rep. 2023 Dec 11;42(12):113494. doi: 10.1016/j.celrep.2023.113494 (PMC10765317; doi:10.1016/j.celrep.2023.113494)
Supplement: Document S1. Figures S1–S12 and Table S1 [file mmc1.pdf]

Cell Reports, Volume *42*

Supplemental information

T cell-mediated curation and restructuring of tumor tissue coordinates an effective immune response

John W. Hickey, Maximillian Haist, Nina Horowitz, Chiara Caraccio, Yuqi Tan, Andrew J. Rech, Marc-Andrea Baertsch, Xavier Rovira-Clavé, Bokai Zhu, Gustavo Vazquez, Graham Barlow, Eran Agmon, Yury Goltsev, John B. Sunwoo, Markus Covert, and Garry P. Nolan

SUPPLEMENTAL FIGURES (1-12) & TABLES (Table 1)

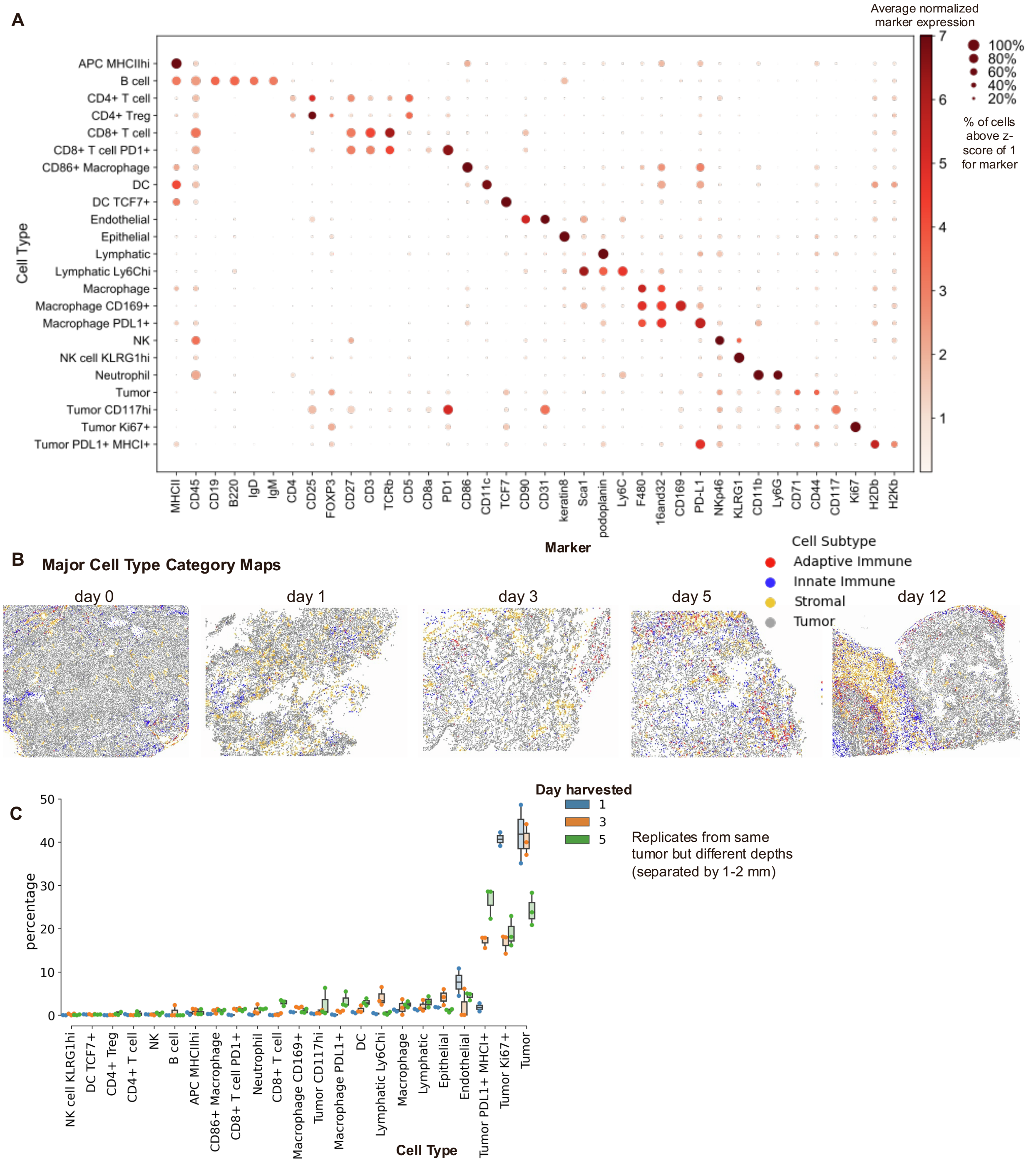

Supplemental Figure 1: Single cell typing for CODEX Multiplexed imaging of T cell treated tumors taken at days 0, 1, 3, 5, or 12 post T cell treatment. **A)** Protein marker expression versus cell type identified for all cell type clusters. Color indicates mean expression of marker by cluster of normalized marker expression. Size indicates percent of cells in this cluster that have a normalized value greater than 1. **B)** Cell type maps for major cell type categories for the tumor images shown in Figure 1 with categories of adaptive immune (red), innate immune (blue), stromal (orange), and tumor (gray). **C)** Low variation in cell type across multiple depths (>mm

apart) for T cell treated tumors taken 1, 3, or 5 days after treatment, as measured by CODEX multiplexed imaging as measured by percentage of each cell type out of total in tumor.

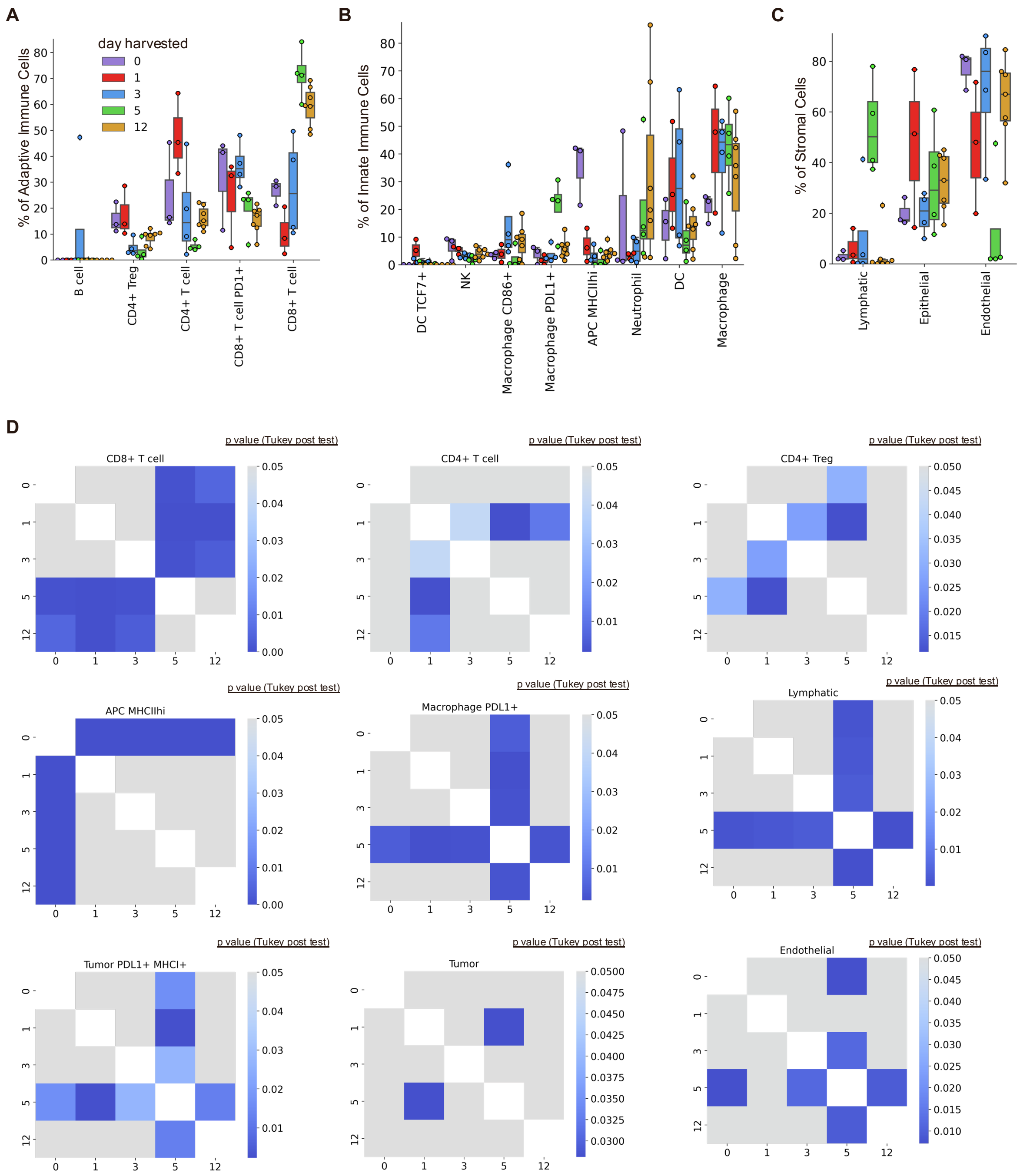

Supplemental Figure 2: Comparisons of cell type proportions across timepoints. **A-C)** Percentages of **A)** adaptive immune, **B)** innate immune, and **C)** stromal cell sub-types normalized to total number of each respective subtype per each sample (n=3-7). **D)** P value heatmaps from Tukey post-test following One-way ANOVA comparisons of cell type proportions across the five timepoints (day 0, 1, 3, 5, 12).

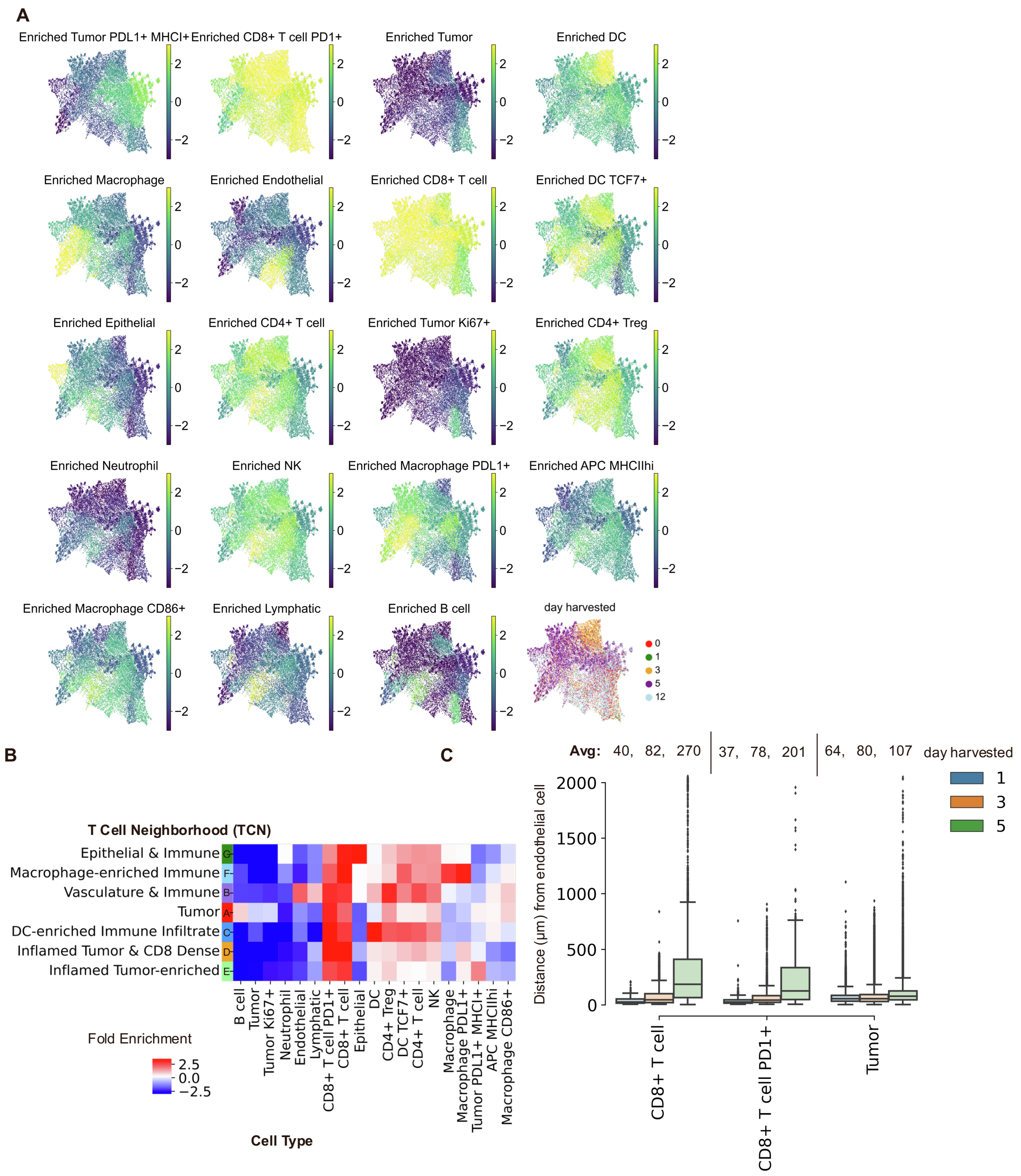

Supplemental Figure 3: T cell specific neighborhoods in tumors across days 0, 1, 3, 5, and 12. **A)** Cell type enrichment for each cell across the spatial UMAP of T cell neighborhoods. **B)** Heat map of the enrichment for cell types within specific neighborhood as compared to tissue averages. **C)** Cell distances for each cell to the nearest endothelial cell in each day harvested with averages plotted over the top.

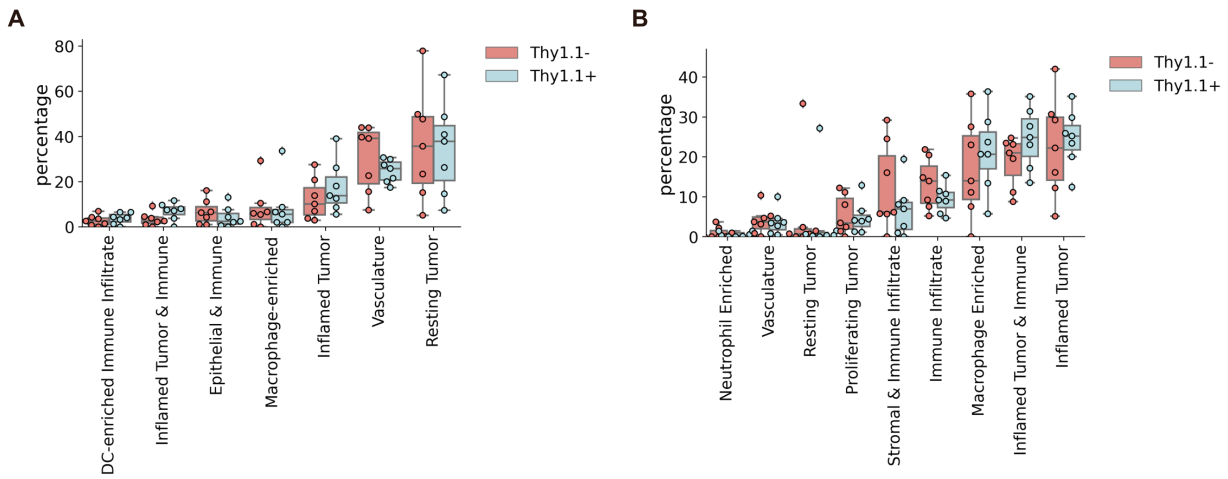

Supplemental Figure 4: Percentages of Thy1.1- vs. Thy1.1+ CD8+ T cell populations. **A-B)** Across either **A)** TCNs or **B)** CNs from tumors of mice taken 12 days after treatment with T cells (n=7).

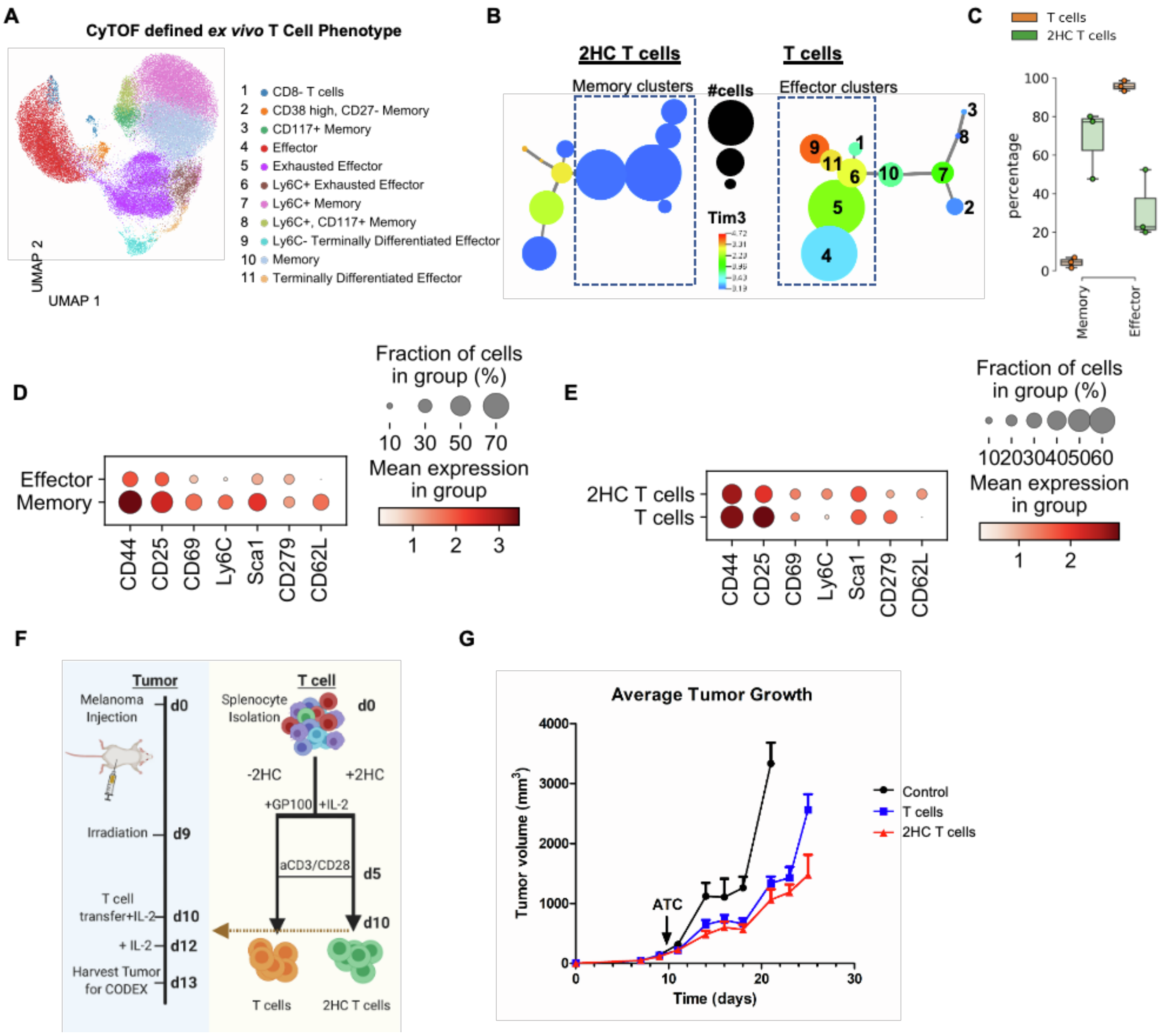

Supplemental Figure 5: Phenotype of treated and untreated activated T cells. **A)** UMAP plot of single cell data of T cells analyzed by a panel of CyTOF antibodies colored by 11 identified clusters. **B)** Minimal spanning trees for the clusters identified by CyTOF in T cells activated in the presence of 2HC (2HC T cells; left) or without 2HC (right). Memory and effector clusters are highlighted. Cluster circle size indicates number of cells in each cluster for each condition and color represents Tim3 average expression within each cluster. **C)** Percentages of memory or effector categories in T cells activated with or without 2HC (n=3 replicates, $p<0.01$). **D)** All cells within all samples were collapsed into two phenotypes and evaluated for average (normalized) marker expression (color) and percent of cells within the cluster above threshold expressing each marker (size). **E)** All cells within all samples were collapsed into treatment categories and evaluated for average (normalized) marker expression (color) and percent of cells within the cluster above threshold expressing each marker (size). **F)** Experimental protocol for testing how differences in T cell phenotype affect the tumor immune response. PMEL T cells were activated with or without 2HC and then used in the adoptive T cell therapy model. Tumors were harvested 3 days after treatment and evaluated by CODEX multiplexed imaging. **G)** Tumor volume growth curve for the three treatment groups where tumors were measured starting at day 7 post-tumor injection and adoptive T cell therapy given on day 10.

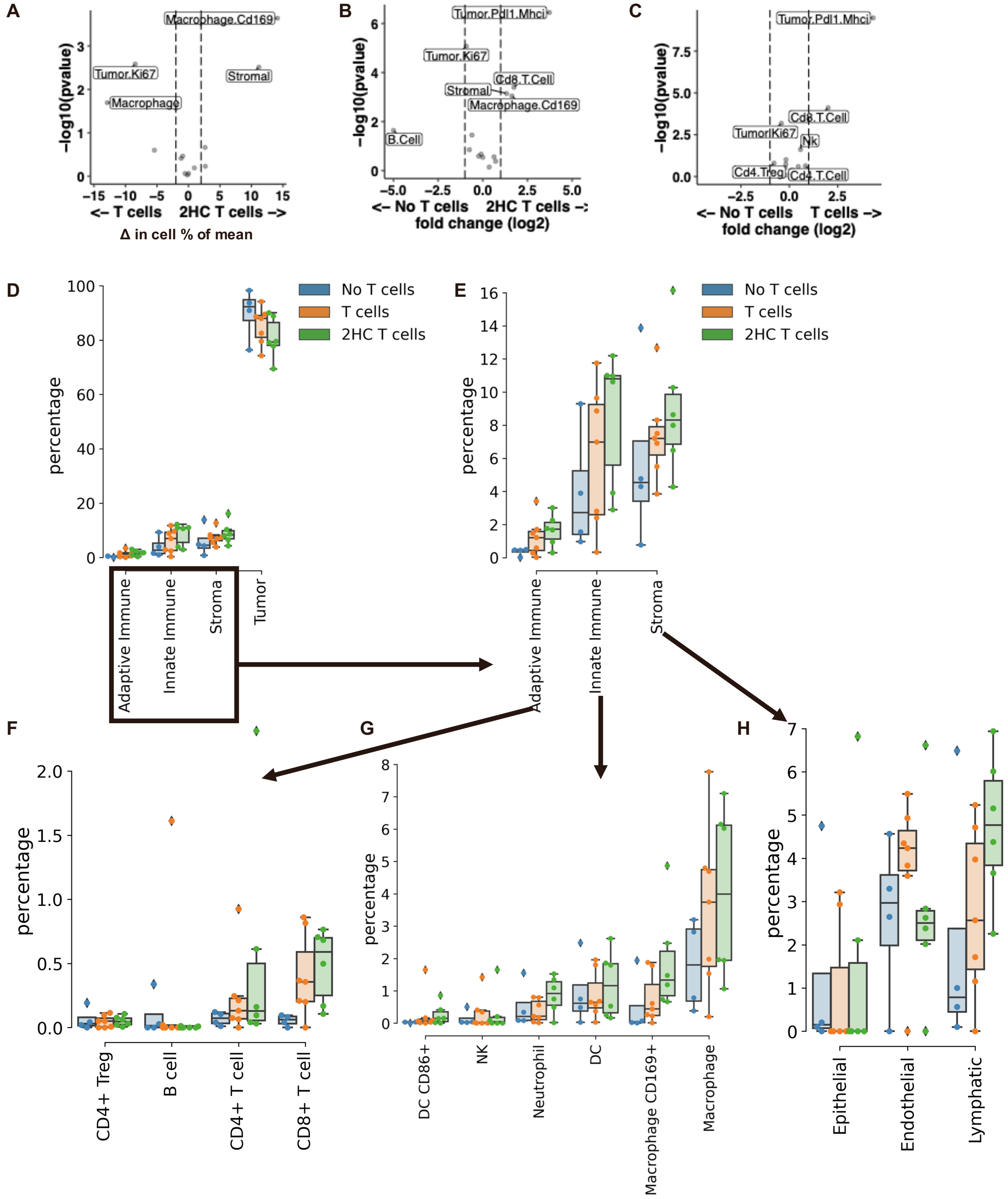

Supplemental Figure 6: Differences in Cell type percentage for CODEX multiplexed imaging data for tumors treated with either 2HC T cells, T cells, or No T cells. **A-C)** Volcano plots presenting an overview of **A)** differences of cell percentage between *2HC T cells and T cells* and **B-C)** fold changes in cell percentage from CODEX imaging data normalized to cell subtype for *No T cells* treated control tumors to **B)** *2HC T cells* or **C)** *T cells* treated tumors. **D)** Percent of

major cell type categories with **E)** showing zoomed in scale without the tumor cell populations. **F)** Percent of adaptive immune cells, **G)** innate immune cells**, H)** stromal cells (n=4-7 replicates).

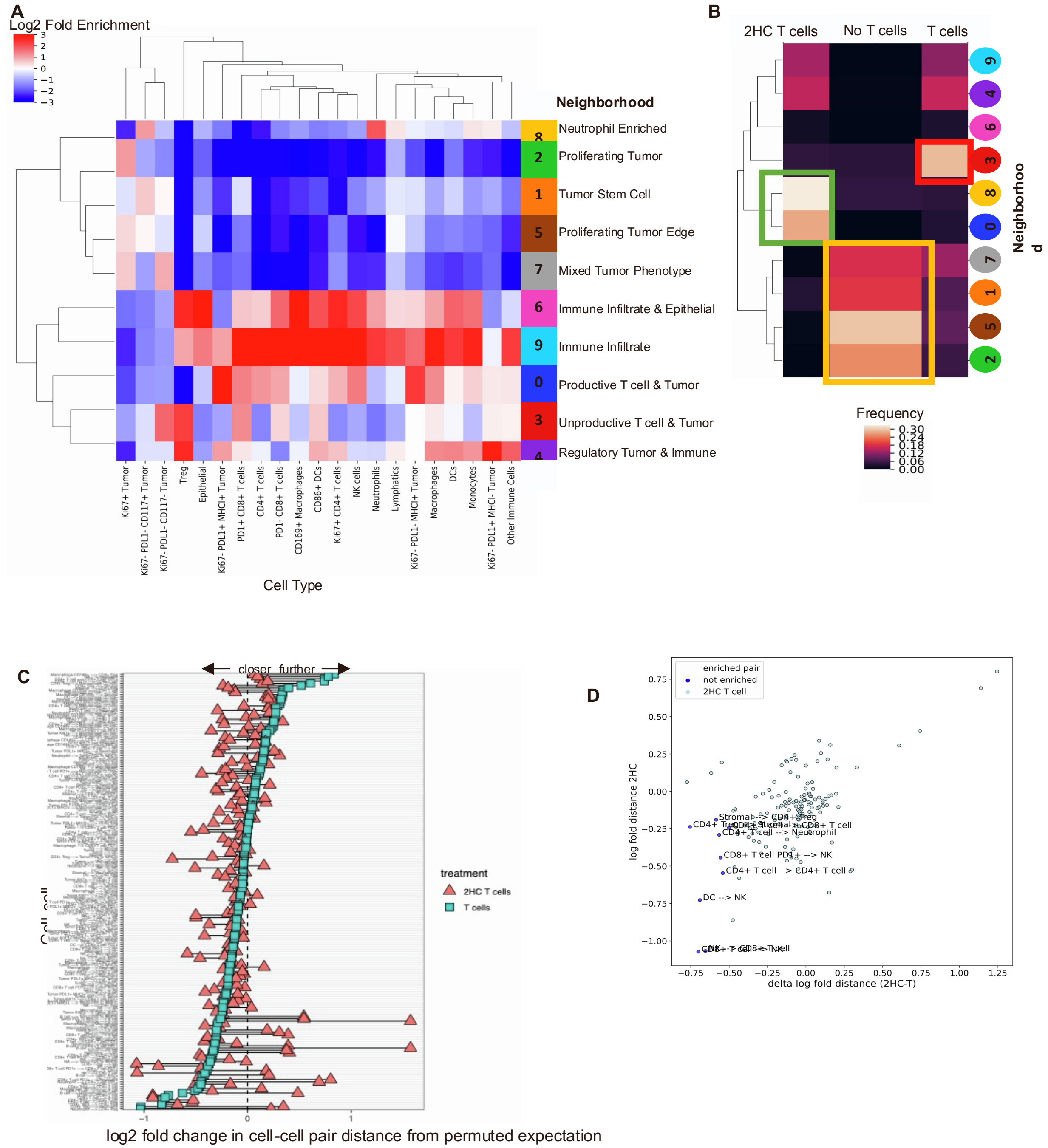

Supplemental Figure 7: Neighborhood and cell-cell interaction analysis of tumors treated with either 2HC T cells, T cells, or No T cells. **A)** Heatmap of neighborhoods (rows) by cell type enrichment (columns) compared to tissue average percentages. **B)** Frequency of cellular neighborhoods among the 3 samples. **C)** All significant cell-cell pair interactions plotted with the log2 fold change of the distance observed between the cell-cell pair in each tumor treatment group compared to permuted controls average. **D)** Subset of the cell-cell pairs plotted in the main figure highlighted in this graph based on a log fold change of the distance for 2HC (y axis) of less than

-0.15 and also a delta of log fold distance (*T cell* subtracted from *2HC T cells*) less than -0.5, indicating cell-cell pair closer especially within *2HC T cells*.

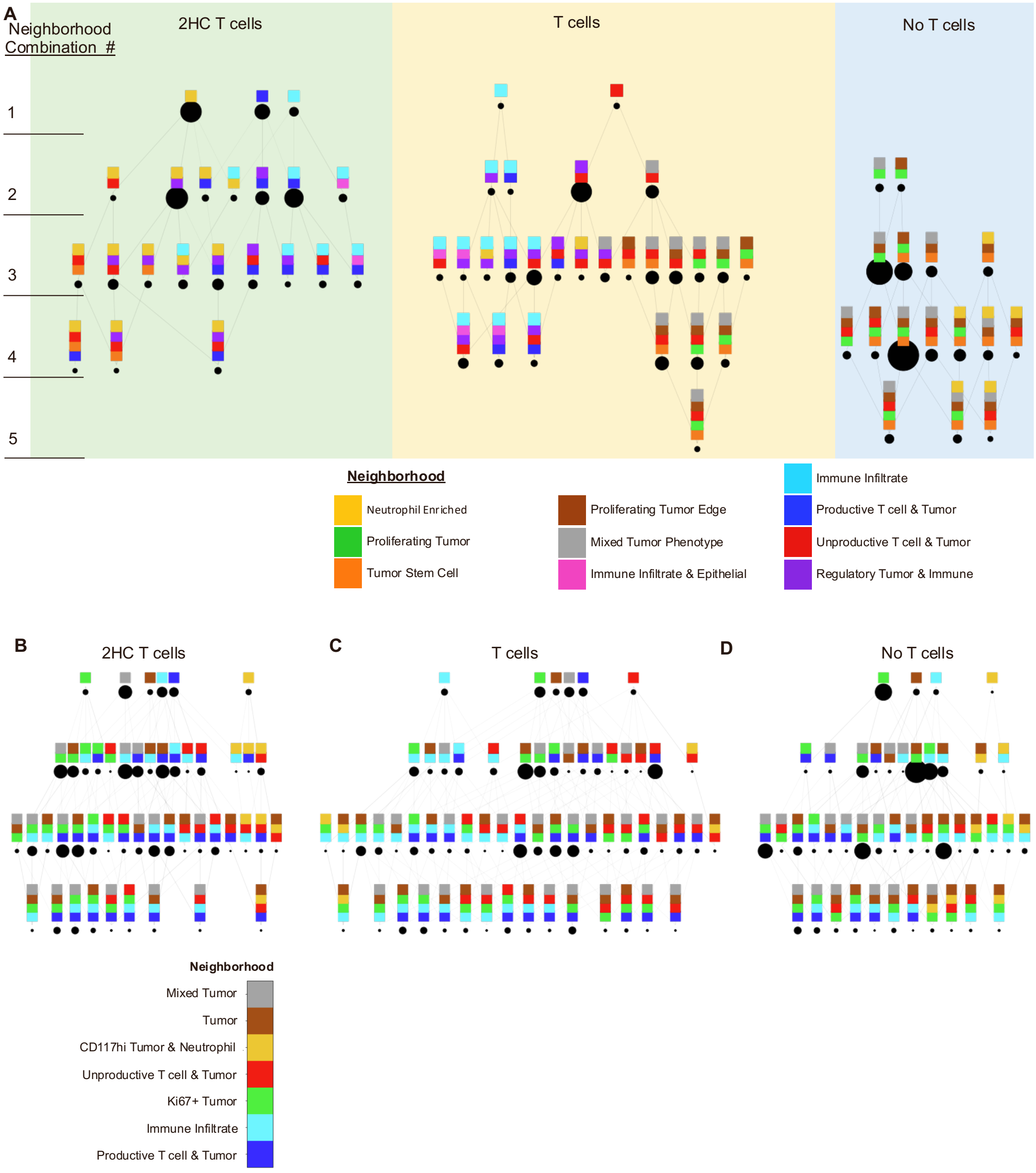

Supplemental Figure 8: Spatial context maps for the tumors treated with either 2HC T cells, T cells, or No T cells. **A)** The first row (#1) shows only one neighborhood which means that this neighborhood alone accounts for more than 85% of the neighborhoods surrounding the window. The second row (#2) shows that this specific combination of 2 neighborhoods makes up more than 85% of the neighborhoods in this window. The third-sixth rows (#3-6) indicate intermixing of multiple neighborhoods. **B-D)** Spatial context maps for all experimental samples combined for **B)** 2HC T cells, **C)** T cells, and **D)** No T cells.

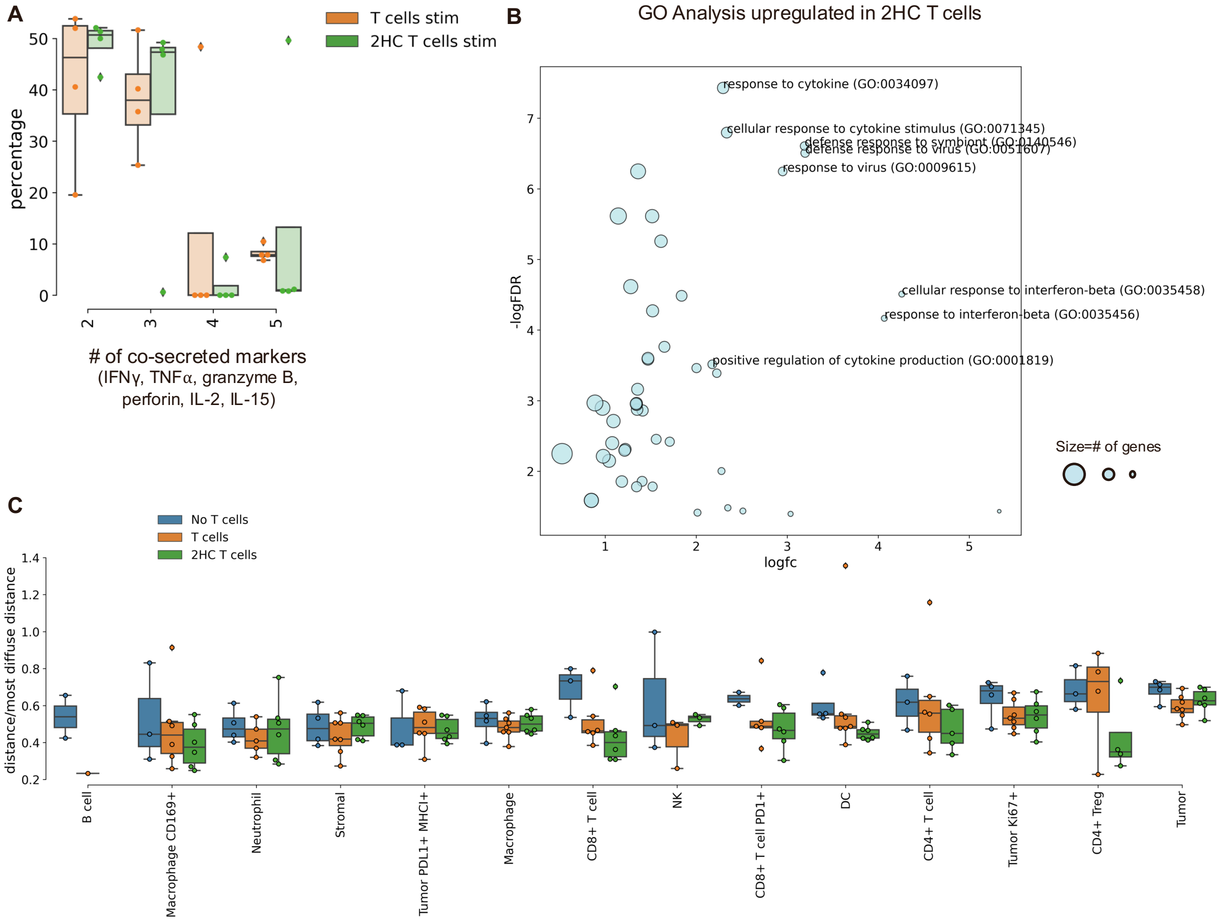

Supplemental Figure 9: Traditional measurements of T cell functionality do not explain differences in T cell *in vivo* efficacy. **A)** Percentage of T cells co-positive for the number of effector molecules (IFNγ, TNFα, granzyme B, perforin, IL-2, IL-15) measured by CyTOF for intracellular staining of restimulated T cells after 10 days of culture (n=4 replicates). **B)** RNA GO analysis for genes expressed higher in *2HC T cells.* ***C*)** Self-same cell density categorized across treatment group samples and cell types. Cell types with less than 5 cells in a tissue imaged were removed from the analysis, hence some cells have less replicates with certain cell types (from an n=4-7).

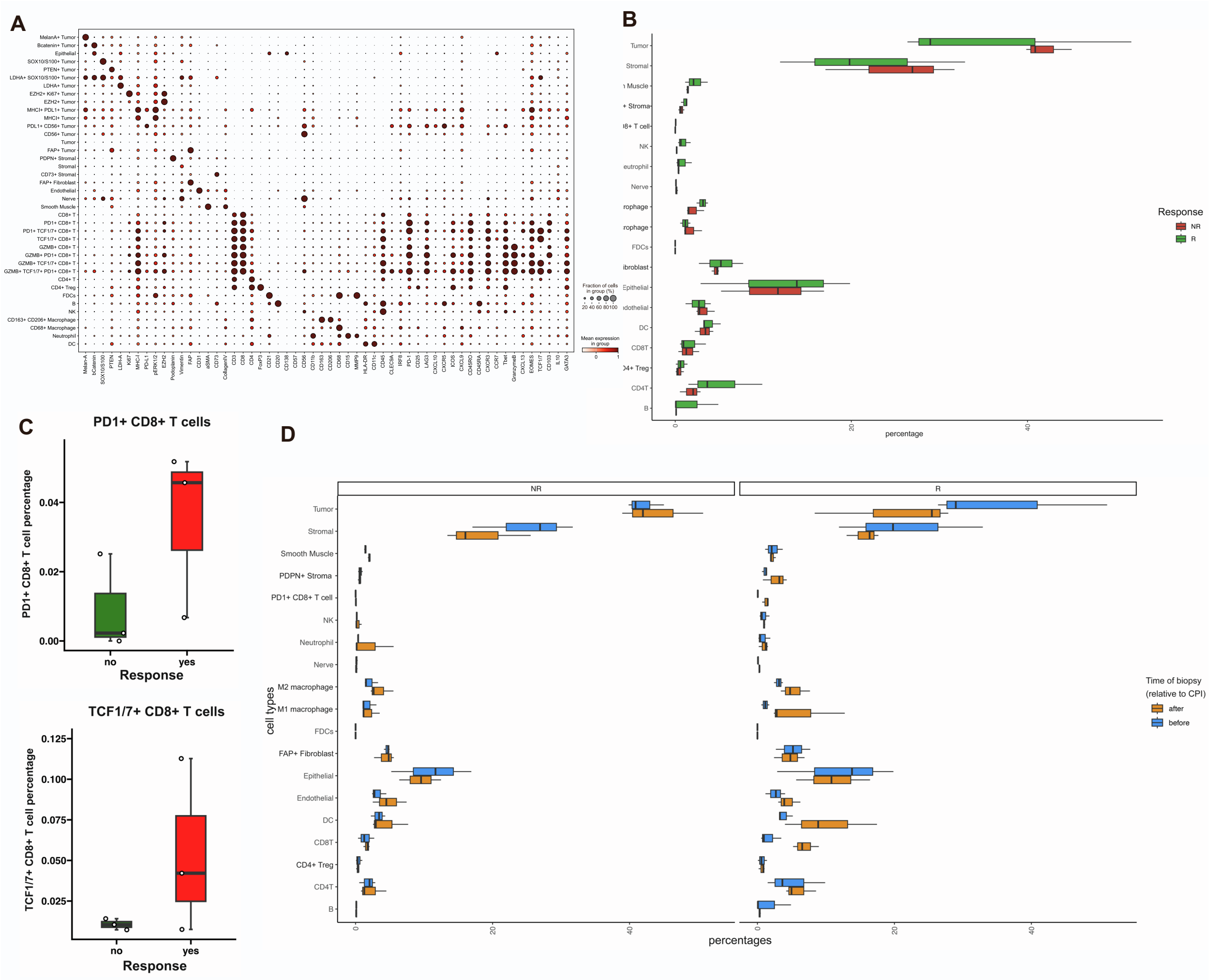

Supplemental Figure 10: Cell type analysis from CODEX multiplexed imaging of melanoma tumors taken from patients before and after checkpoint inhibition therapy. **A)** Protein marker expression versus cell type identified for all cell type clusters. Color indicates mean expression of marker by cluster of normalized marker expression. Size indicates percent of cells in this cluster that have a normalized value greater than 1. **B)** Cell type percentage averages from either non-responders (NS) or responders (R) from coarse cell type classifications. **C)** Percentage of different T cell phenotypes with tumors from patients that had a response to therapy or did not. **D)** Percentages of coarse cell types separated by both NR and R and also before and after therapy. (For all panels: n=6 patients and 12 tumors imaged).

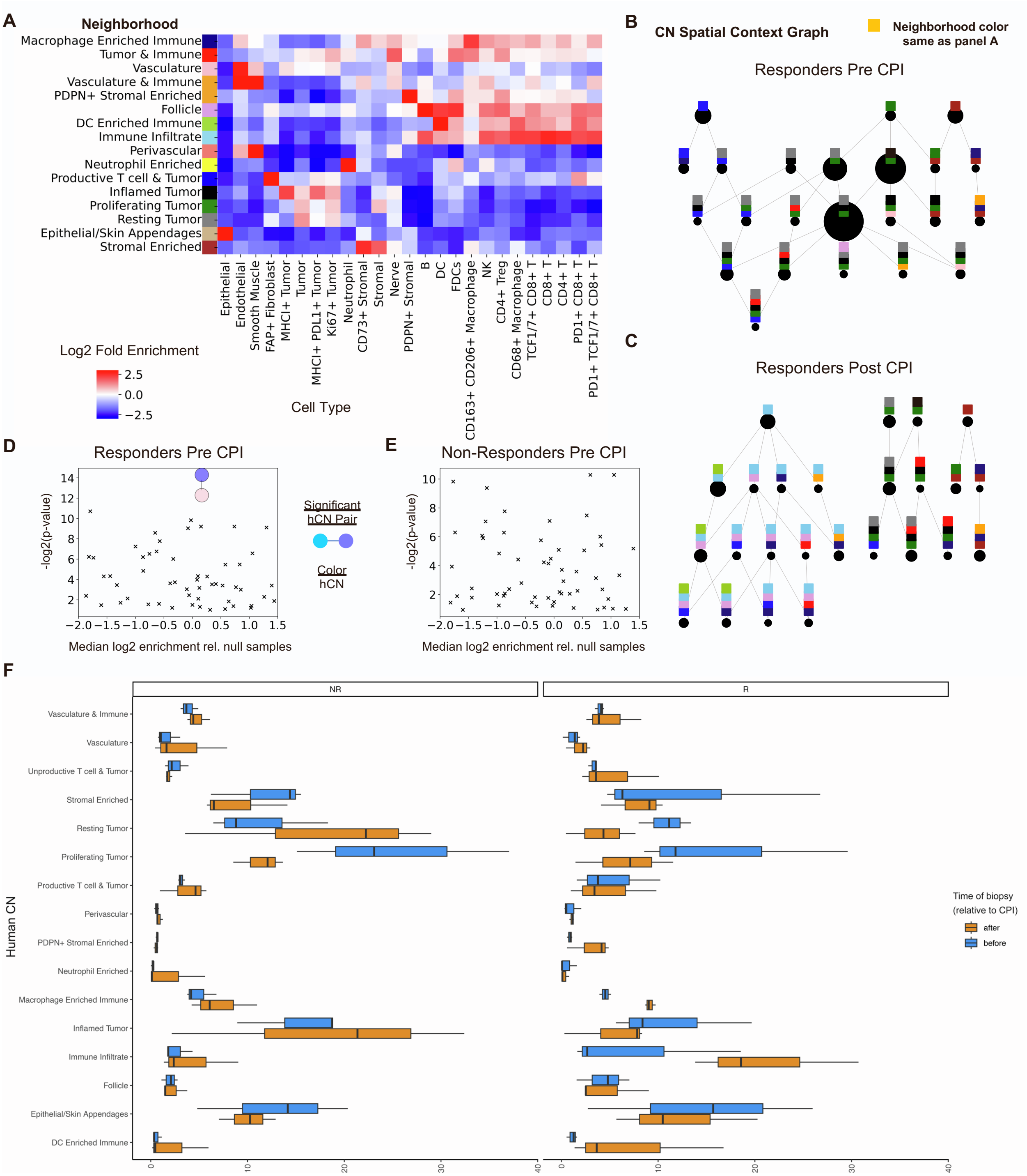

Supplemental Figure 11: Spatial analyses of CODEX multiplexed imaging data of melanoma tumors taken from patients before and after checkpoint inhibition therapy. **A)** Heatmap of neighborhoods (rows) by cell type enrichment (columns) compared to tissue average percentages. **B-C)** Spatial context maps for **B)** responders before checkpoint blockade therapy and **C)** after checkpoint blockade therapy. **D-E)** Significant two-combination neighborhood motifs enriched or depleted compared to null distribution of permuted samples. Color represents shared neighborhood found across all samples. Only neighborhoods forming a significant connection with

the *Productive T cell & Tumor* hCN are shown. For tumors taken prior to therapy for either **D)** responders or **E)** non-responders. **F)** Quantitative comparisons for the abundance of each human melanoma cellular neighborhood stratified by Responder (R, right) and Non-responder (NR, left) and time of tumor biopsy (blue: before treatment start; orange: after start of therapy) (For all panels: n=6 patients and 12 tumors imaged)

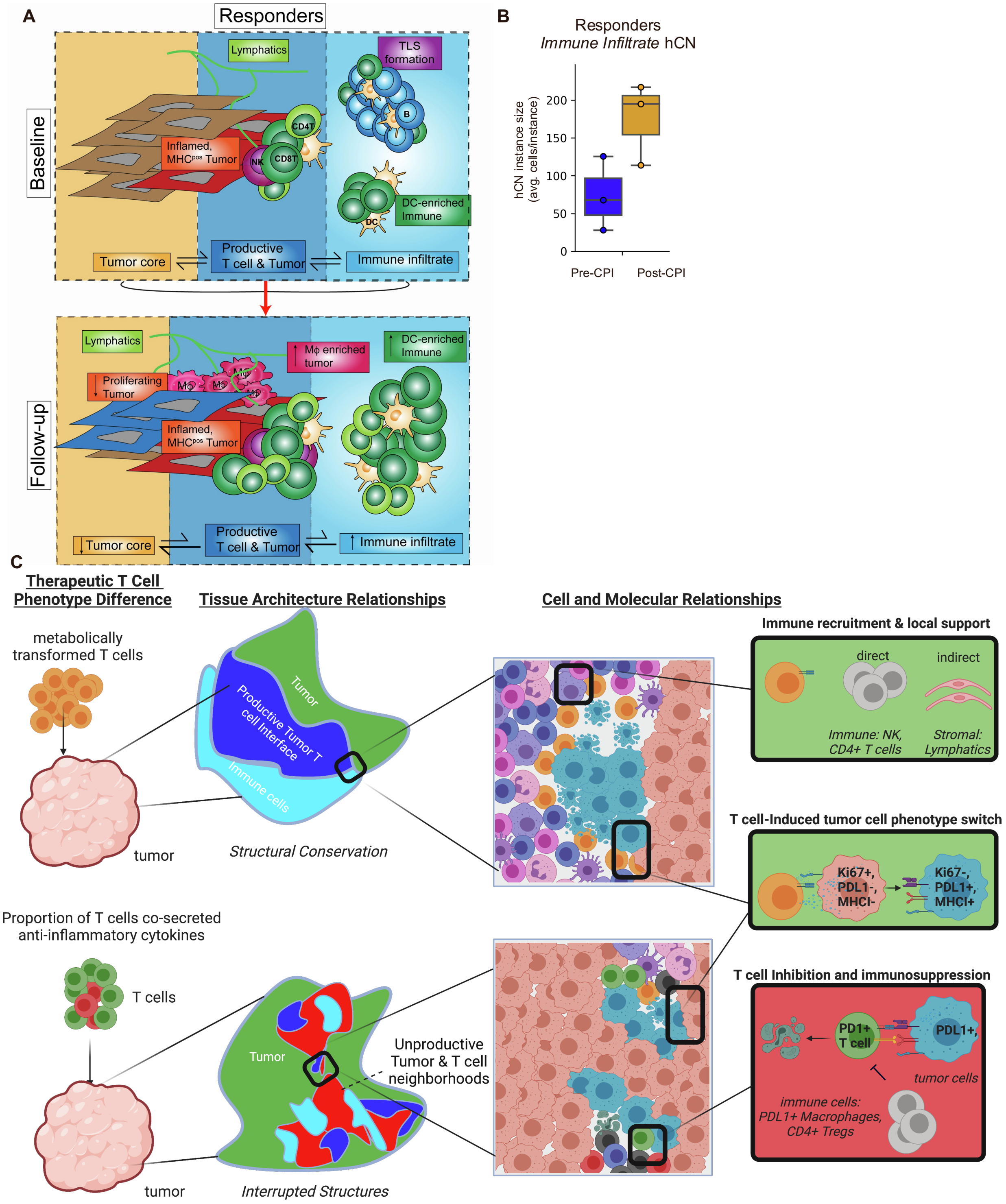

Supplemental Figure 12: Major cellular and organizational changes are observed within tumors of patients who respond to checkpoint blockade therapy. **A)** Overall diagram of the cellular and cellular neighborhood changes within responders to checkpoint blockade at baseline (prior to treatment) versus at follow-up (post-treatment). **B)** Size of the instance for the *Immune Infiltrate* human cellular neighborhood (hCN) as measured by the average number of cells per instance

from the responders' tumors pre-therapy and post-therapy. (For all panels: n=6 patients and 12 tumors imaged, and n=3 for responders and n=3 for non-responders). **C)** T cell phenotype impacts the ability of T cells to create productive tumor T cell killing interfaces. We found here that traditionally activated T cells have a proportion of T cells that also expressed anti-inflammatory cytokines that result in tumors in which productive T cell and tumor neighborhoods are disrupted by regulatory neighborhoods. Treating tumors with metabolically manipulated T cells resulted in larger structural compartmentalization of productive tumor and T cell neighborhoods that co-associate with areas of immune infiltration. Both T cell treatments led to a tumor phenotype conversion that was associated with inflammatory marker upregulation and inhibition of proliferation. T cells in human patient melanomas that responded to checkpoint blockade therapy also had compartmentalization and colocalization of dense immune infiltration cellular neighborhoods next to zones where T cells were engaging tumors. Consequently, therapeutic T cells should be designed for their capacity to kill tumor cells, convert tumor cell phenotype, secrete only inflammatory cytokines, maintain phenotype, and recruit immune cells.

Supplementary Table 1: Baseline patient characteristics of the melanoma cohort investigated in this study.

Clinicopathological characteristics	CPI responders	CPI non-responders
Total number of patients	3	3
Age at start of CPI therapy	71.3 (46.2-96.5)	67.3 (61.1-63.6)
Gender<br>- Male<br>- Female	 2<br>1	 1<br>2
**Primary tumor characteristics**		
Mean tumor thickness (95% CI)	4.4 (0-16.5)	4.7 (3.6-5.9)
Ulceration[1]	2	2
BRAF mutation status<br>- BRAFV600 positive<br>- BRAFV600 negative	 2<br>1	 1<br>2
AJCC stage at initial diagnosis<br>- IIB<br>- IIIC	 1<br>2	 0<br>3
Mean baseline serum LDH levels in IU/ml	239 (0-531)	408 (0-1189)
**Treatments for metastatic stage IV disease**		
1L treatments<br>- cICB<br>- Pembrolizumab plus dabrafenib/trametinib<br>- Nivolumab	 2<br>1<br>0	 2<br>0<br>1
Mean treatment duration (95% CI)	14.3 (4.3-24.4)	2.3 (0.9-3.8)
Best overall response<br>- PD<br>- SD<br>- PR<br>- CR	 0<br>0<br>0<br>3	 3<br>0<br>0<br>0
Tumor progress	2	3
Median PFS (95% CI)	23 months (5-NR)	2 months (2-NA)
2L treatments<br>- Radiotherapy<br>- BRAF/MEKi<br>- Study<br>- Pembrolizumab<br>- Chemotherapy (DTIC)	 1<br>1<br>0<br>1<br>0	 0<br>1<br>1<br>0<br>1
**Survival and follow-up**		

Median follow-up (95% CI)	50.0 months (23.0-NR)	NR (9.0-NR)
Deceased	0	2
Median OS (95% CI)	Not reached	32.5 months (26.0-NR)

[1] Ulceration status was known in 4/6 patients.

Abbreviations: 1L = first-line; 2L = second-line; BRAF/MEKi = BRAF/MEK-inhibitors; CPI = checkpoint-inhibitor therapy; CI = confidence interval; CPI = checkpoint-inhibitor therapy; CR = complete response; DTIC = dacarbazine; LDH = lactate dehydrogenase; NR = not reached; PD = progressive disease; SD = stable disease
